# Aerosol responses to precipitation along North American air trajectories arriving at Bermuda

**DOI:** 10.5194/acp-21-16121-2021

**Published:** 2021-11-02

**Authors:** Hossein Dadashazar, Majid Alipanah, Miguel Ricardo A. Hilario, Ewan Crosbie, Simon Kirschler, Hongyu Liu, Richard H. Moore, Andrew J. Peters, Amy Jo Scarino, Michael Shook, K. Lee Thornhill, Christiane Voigt, Hailong Wang, Edward Winstead, Bo Zhang, Luke Ziemba, Armin Sorooshian

**Affiliations:** 1 Department of Chemical and Environmental Engineering, University of Arizona, Tucson, AZ, USA; 2 Department of Systems and Industrial Engineering, University of Arizona, Tucson, AZ, USA; 3 Department of Hydrology and Atmospheric Sciences, University of Arizona, Tucson, AZ, USA; 4 NASA Langley Research Center, Hampton, VA, USA; 5 Science Systems and Applications, Inc., Hampton, VA, USA; 6 Institute for Atmospheric Physics, DLR, German Aerospace Center, Oberpfaffenhofen, Germany; 7 Institute for Atmospheric Physics, University of Mainz, Mainz, Germany; 8 National Institute of Aerospace, Hampton, VA, USA; 9 Bermuda Institute of Ocean Sciences, 17 Biological Station, St. George’s, GE01, Bermuda; 10 Atmospheric Sciences and Global Change Division, Pacific Northwest National Laboratory, Richland, WA, USA

## Abstract

North American pollution outflow is ubiquitous over the western North Atlantic Ocean, especially in winter, making this location a suitable natural laboratory for investigating the impact of precipitation on aerosol particles along air mass trajectories. We take advantage of observational data collected at Bermuda to seasonally assess the sensitivity of aerosol mass concentrations and volume size distributions to accumulated precipitation along trajectories (APT). The mass concentration of particulate matter with aerodynamic diameter less than 2.5 μm normalized by the enhancement of carbon monoxide above background (PM_2.5_/ΔCO) at Bermuda was used to estimate the degree of aerosol loss during transport to Bermuda. Results for December–February (DJF) show that most trajectories come from North America and have the highest APTs, resulting in a significant reduction (by 53 %) in PM_2.5_/ΔCO under high-APT conditions (> 13.5 mm) relative to low-APT conditions (< 0.9 mm). Moreover, PM_2.5_/ΔCO was most sensitive to increases in APT up to 5 mm (−0.044 μg m^−3^ ppbv^−1^ mm^−1^) and less sensitive to increases in APT over 5 mm. While anthropogenic PM_2.5_ constituents (e.g., black carbon, sulfate, organic carbon) decrease with high APT, sea salt, in contrast, was comparable between high- and low-APT conditions owing to enhanced local wind and sea salt emissions in high-APT conditions. The greater sensitivity of the fine-mode volume concentrations (versus coarse mode) to wet scavenging is evident from AErosol RObotic NETwork (AERONET) volume size distribution data. A combination of GEOS-Chem model simulations of the ^210^Pb submicron aerosol tracer and its gaseous precursor ^222^Rn reveals that (i) surface aerosol particles at Bermuda are most impacted by wet scavenging in winter and spring (due to large-scale precipitation) with a maximum in March, whereas convective scavenging plays a substantial role in summer; and (ii) North American ^222^Rn tracer emissions contribute most to surface ^210^Pb concentrations at Bermuda in winter (~75 %–80 %), indicating that air masses arriving at Bermuda experience large-scale precipitation scavenging while traveling from North America. A case study flight from the ACTIVATE field campaign on 22 February 2020 reveals a significant reduction in aerosol number and volume concentrations during air mass transport off the US East Coast associated with increased cloud fraction and precipitation. These results highlight the sensitivity of remote marine boundary layer aerosol characteristics to precipitation along trajectories, especially when the air mass source is continental outflow from polluted regions like the US East Coast.

## 1 Introduction

Aerosol properties are difficult to characterize in remote marine regions owing to the scarcity of monitoring stations compared to over land. Island observatories are critical resources to investigate long-range transport of aerosol particles and their associated properties (e.g., Silva et al., 2020). The western North Atlantic Ocean (WNAO) includes the island of Bermuda, which has a rich history of monitoring data for both surface and columnar aerosol characteristics, thus affording the opportunity to study how aerosol properties are impacted by different sources and processes along the transport of air masses to the site. Consequently, Bermuda has been the subject of decades of intense atmospheric science research (Sorooshian et al., 2020), as it is a receptor site for both North African dust (Chen and Duce, 1983) and anthropogenic outflow from both North America (Arimoto et al., 1992; Galloway et al., 1989; Moody et al., 2014; Corral et al., 2021) and Europe (Anderson et al., 1996; Cutter, 1993). North American outflow reaching Bermuda has been linked to appreciable levels of anthropogenic species (e.g., sulfate, lead, elemental carbon, ozone) (Wolff et al., 1986), more acidic rainfall compared to other air mass sources (Jickells et al., 1982), and a significant reduction of sulfate levels in both aerosol and wet deposition samples in response to reduced SO_2_ emissions in recent decades (Keene et al., 2014).

There have been extensive studies reporting on some aspect of air mass history, normally by calculating air parcel trajectories using transport and dispersion models, prior to arrival at Bermuda (Sorooshian et al., 2020, and references therein), including predominant circulation patterns impacting Bermuda at different times of the year (e.g., Miller and Harris, 1985; Veron et al., 1992). What remains uncertain is how precipitation along those trajectories impacts surface aerosol characteristics at Bermuda. Wet scavenging rates are very difficult to constrain over open-ocean areas such as the WNAO (Kadko and Prospero, 2011) not only because of the complexity of physical mechanisms in play but also scarce necessary field measurements. Overall, more work is warranted to better constrain wet scavenging of aerosol particles along trajectories as such studies are sparse not only for the WNAO but also for other regions (Tunved et al., 2013; Hilario et al., 2021). Arimoto et al. (1999) used aerosol radionuclide data in relation to airflow pattern information to conclude that pollutant transport to Bermuda is common from the northwest and that precipitation scavenging can be influential; their analysis of rain effects on nuclide activities were based on rain data collected at Bermuda without knowledge of precipitation transport history prior to arrival. While many studies have investigated how composition at Bermuda varies based on air mass trajectories (Miller and Harris, 1985; Cutter, 1993; Huang et al., 1996), the subject of how precipitation along those trajectories impacts the resultant aerosol at Bermuda has not been adequately addressed but is motivated by past works (Moody and Galloway, 1988; Todd et al., 2003).

In their recent aerosol climatology study for Bermuda, Aldhaif et al. (2021) found the peculiar result that fine particulate pollution in the winter months (December–February) was reduced even though there was an enhanced number density of air mass back trajectories traced back to North America. They hypothesized that enhanced seasonal cloud fractions and precipitation in winter (Painemal et al., 2021) contribute to the removal of aerosol particles during transport via wet scavenging, which we aim to study more deeply here using a variety of datasets. Results of this study have broad relevance to all remote marine regions impacted by transported continental pollution, in addition to advancing knowledge of how precipitation can impact surface aerosol characteristics.

## 2 Datasets and methods

Datasets used in this work are summarized in Table 1 and briefly described below.

### 2.1 Bermuda surface measurements

Aerosol and gas measurements were conducted at Fort Prospect in Bermuda (32.30° N, 64.77° W, 63ma.s.l.). Hourly PM_2.5_ data were collected with a Thermo Scientific TEOM 1400a ambient particulate monitor with 8500C FDMS (Federal Equivalent Method EQPM-0609-181 for PM_2.5_). Concentrations were determined by employing conditioned filter sample collection and direct mass measurements using an inertial micro-balance (TEOM 1400a). Hourly precision was ±1.5 μgm^−3^. Hourly data were averaged over 6 h intervals to match the time frequency of the trajectory data discussed subsequently. The conversion of hourly data to 6 h data also helps to mask, to some extent, the unwanted effects of local sources and processes that occur on a small timescale.

PM_10_ concentrations were determined based on US Environmental Protection Agency (EPA) method IO-2 (EPA, 1999) using a Tisch model TE6070 hi-volume air sampler, equipped with 8″ × 10″ (20.3 cm × 25.4 cm) TissuQuartz 2500 QAT-UP quartz-fiber filters. The PM_10_ sampler was operated at a flow rate of 2.1 m^3^ min^−1^, yielding a total volume of 3000 m^3^ over a 24 h sampling period. The sampler flow rate was calibrated every 3 months. Sampling was synchronized with the 1-in-6 d national ambient air quality schedule used by the EPA. Prior to deployment, the filters were equilibrated for 24 h in an environmental control chamber maintaining constant conditions of relative humidity (35 ± 2 %) and temperature (21 ± 2 °C). The filters were then weighed with a precision of ±0.1 mg using a Mettler Toledo AB104 balance, which was modified for weighing unfolded 8″ × 10” (20.3 cm × 25.4 cm) filters, and then transferred to clean resealable plastic bags for transportation to the field site. After sampling, the exposed filters were returned immediately to the laboratory where they were re-equilibrated in the environmental control chamber for 24 h before being reweighed to determine the particle loading from which particle concentrations were calculated. PM_10_ determinations have an accuracy of within ±2.5 %, which is equivalent to ±0.2 μgm^−3^ based on the average of PM_2.5_ between 2015 and 2019 (i.e., 6.7 μgm^−3^).

Various gases were monitored with hourly time resolution using a model T200U trace-level NO/NO_2_/NO*_x_* analyzer (Teledyne API), which is a US EPA compliance analyzer relying on a proven chemiluminescence principle. The gas analyzer was routinely calibrated using NIST-certified calibrant NO_2_ in ultrahigh-purity nitrogen (Airgas, Inc., Radnor Township, PA, USA). Acceptable criteria applied for single-point quality control (QC) allow for ±15.1 % or < ±1.5 ppb difference, whichever is greater (40 CFR Part 58 App A Sect. 3.1.1). Similar to PM_2.5_, these hourly gas data were averaged to 6 h resolution.

There were a few periods when data were missing, with the longest one being between 11 January and 8 April 2016 for the gases and also between 16 October 2017 and 20 January 2018 for PM_2.5_. There was no major discontinuity in PM_10_ sampling. Table S1 in the Supplement reports the number of data points available for various seasons from the surface measurements at Fort Prospect in Bermuda.

Columnar aerosol data were obtained from a NASA AErosol RObotic NETwork (AERONET) (Holben et al., 1998) surface station at Tudor Hill (32.264° N, 64.879° W). Level 2 daily data have been quality-assured and cloud-screened based on the version 3 algorithm (Giles et al., 2019). We focus on the volume size distribution (VSD) product that has 22 logarithmically equidistant discrete radii ranging from 0.05 to 15 μm. A radius of 0.6 μm typically discriminates between fine and coarse modes when using AERONET data (Dubovik et al., 2002; Schuster et al., 2006).

## 3 Reanalysis data

Modern-Era Retrospective analysis for Research and Applications-Version 2 (MERRA-2) (Gelaro et al., 2017) products were used as a data source for speciated aerosol and gas parameters including surface mass concentration of sea salt (collection tavg1_2d_aer_Nx) and surface concentration of carbon monoxide (CO; collection tavg1_2d_chm_Nx). Surface wind speed and planetary boundary layer height (PBLH) (collection tavg1_2d_flx_Nx) data were also obtained from MERRA-2. Hourly and 3-hourly data were downloaded and averaged for a 0.5° latitude by 0.625° longitude grid (i.e., 32–32.5° N and 64.375–65° W) surrounding Bermuda and subsequently averaged over 6 h intervals to match the time frequency of trajectory analysis results. It should be noted that MERRA-2 data were temporally and spatially coincident with the ending point of trajectories over Bermuda. The Global Data Assimilation System (GDAS) 1° archive data were used for trajectory calculations explained in the subsequent section. Precipitation data were also obtained along the trajectories based on GDAS 1° data.

### 3.1 Air mass trajectory analysis

**Figure 1.**
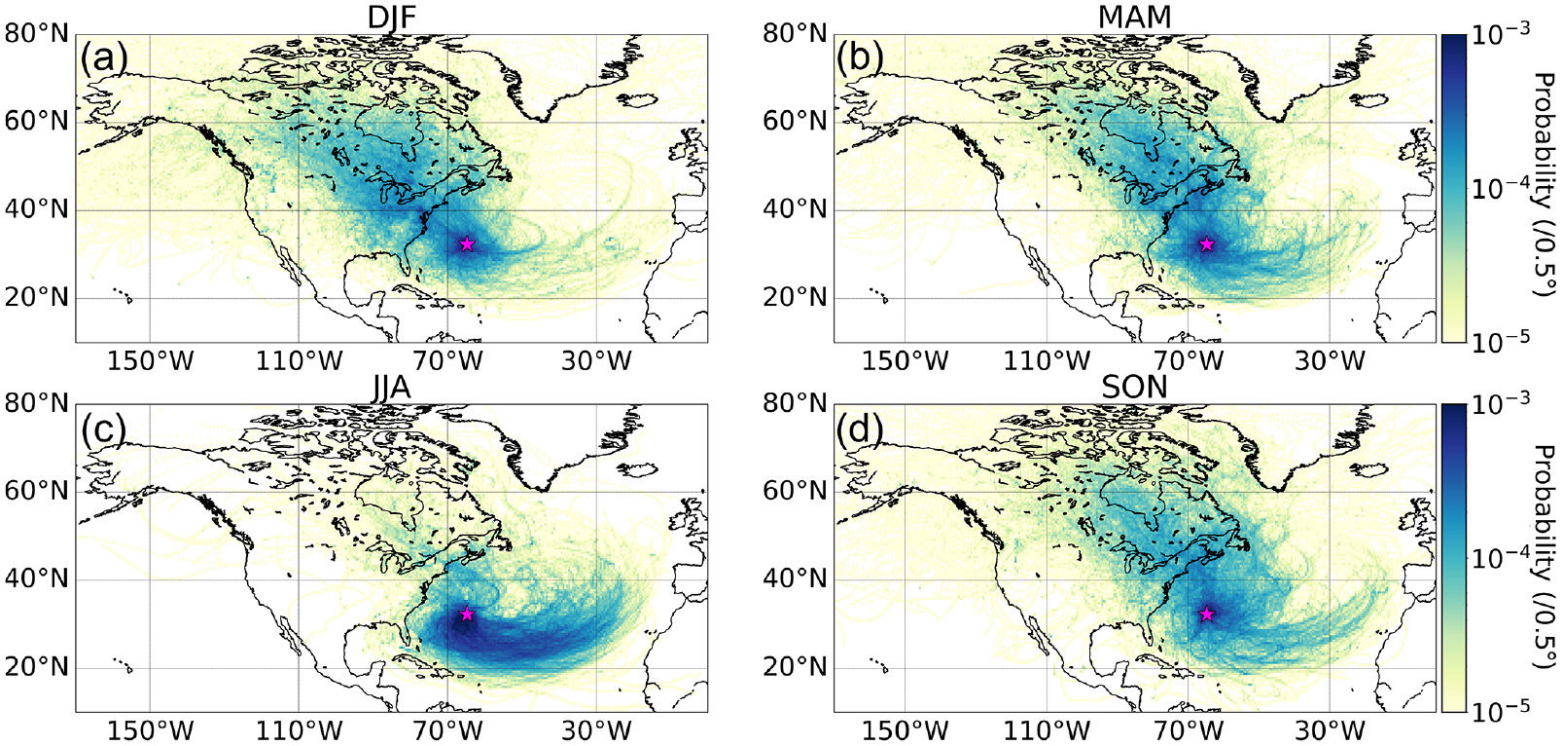
Seasonal maps **(a–d)** showing the probability density of trajectories calculated based on 10 d HYSPLIT backward trajectories reaching Bermuda (32.30° N, 64.77° W), denoted by the pink star, at 100 m (a.g.l.). This analysis is based on trajectories between 1 January 2015 and 31 December 2019. Analogous results for ending altitudes of 500m and 1 km are shown in Figs. S1 and S2, respectively.

**Figure 2.**
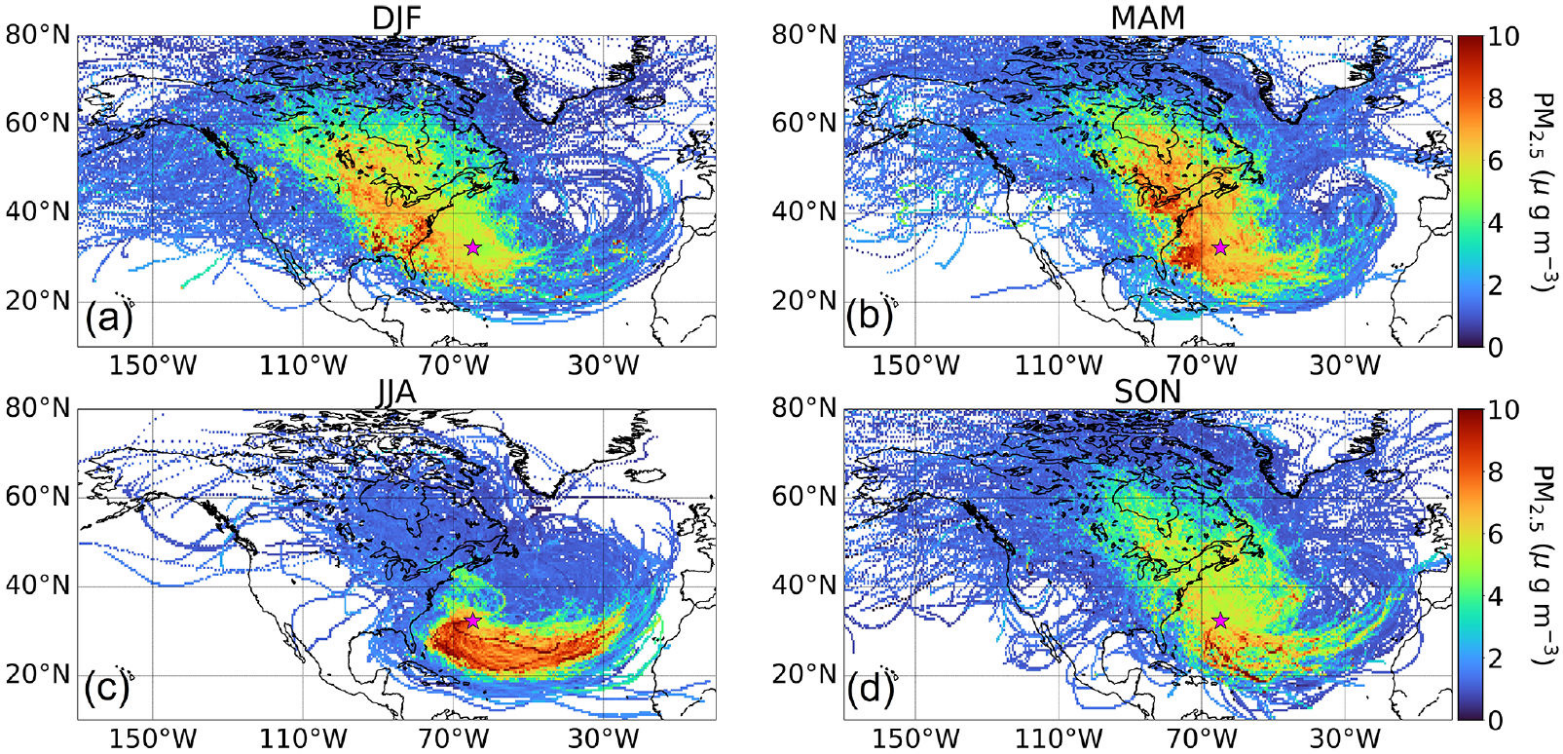
Seasonal **(a–d)** concentration-weighted trajectory maps (CWTs) for PM_2.5_ measured at Fort Prospect in Bermuda, denoted by the pink star. This analysis is based on trajectories between 1 January 2015 and 31 December 2019.

**Figure 3.**
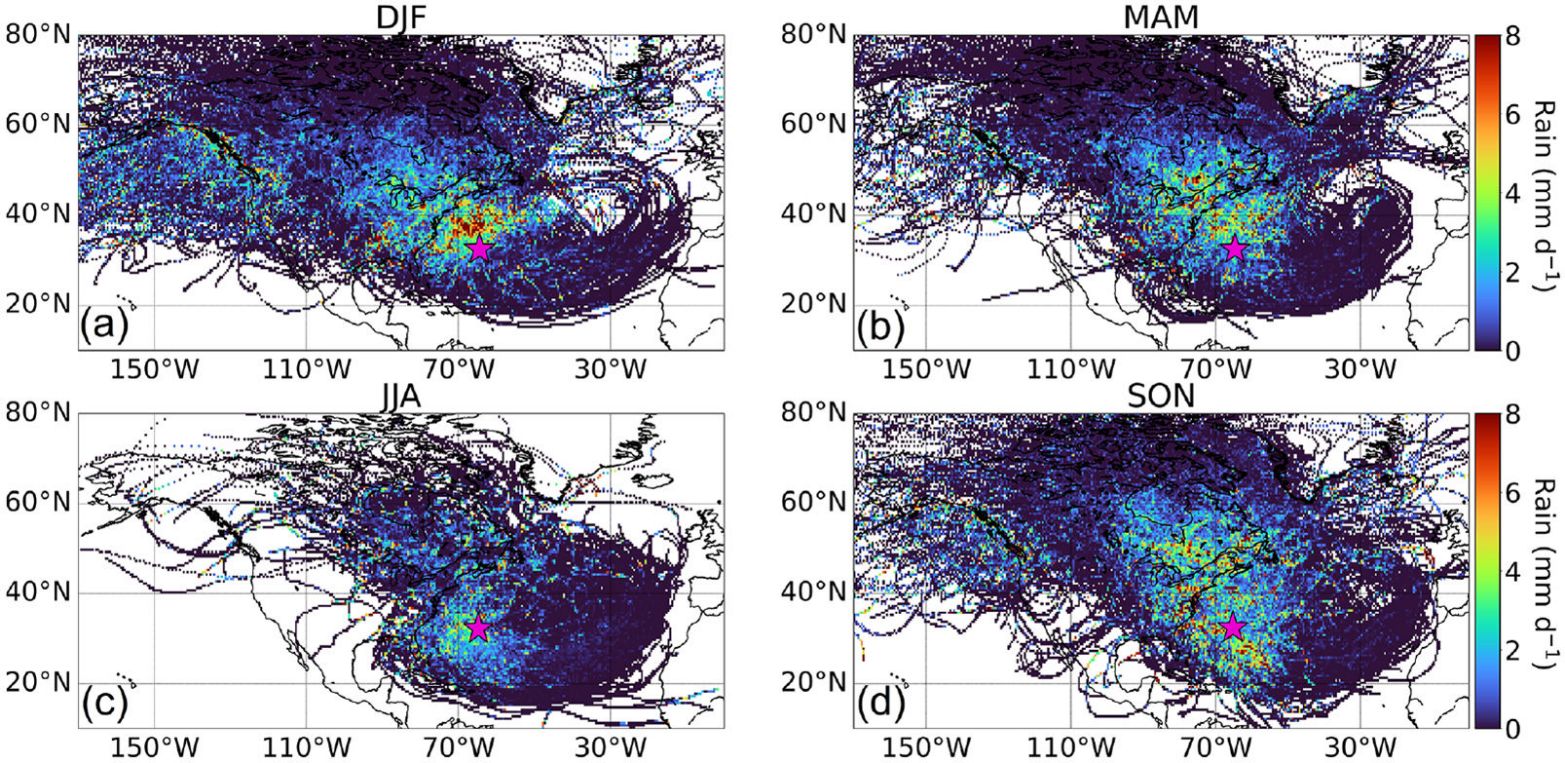
Seasonal maps **(a–d)** of average precipitation occurring in 0.5° × 0.5° grids based on 10 d backward trajectories reaching Bermuda (32.30° N, 64.77° W; pink star) at 100 m (a.g.l.). This analysis is based on trajectories between 1 January 2015 and 31 December 2019.

To track air mass pathways arriving at Bermuda (32.30° N, 64.77° W), we obtained 10 d (240 h) back trajectories from the Hybrid Single-Particle Lagrangian Integrated Trajectory model (HYSPLIT) (Stein et al., 2015; Rolph et al., 2017). We used an ending altitude of 100 m (a.g.l.) to be within the surface layer and close to the measurement site. As discussed later, sensitivity analysis with higher ending altitudes (500 m and 1 km; Figs. S1-S2 in the Supplement) reveals results similar to using 100 m. Four trajectories were initialized (i.e., 6 h interval) each day between 1 January 2015 at 00:00:00 UTC and 31 December 2019 at 18:00:00 UTC, resulting in a total of 7304 individual trajectories. Trajectories were calculated using the GDAS 1° archive data and with the “model vertical velocity” method, which means vertical motions were handled directly using meteorological data files. Moreover, accumulated precipitation along trajectories (APT) was calculated by integrating surface precipitation data from GDAS throughout the transport to the receptor site. As GDAS precipitation data correspond to the surface level, it should be noted that APT values presented in this study are associated with the potential maximum level of precipitation experienced by the air parcel through its transport journey. Results presented in Figs. 1-3 are based on 10d back trajectories, whereas analyses presented in the remaining sections of the paper are based on 4 d (96 h) back trajectories.

Trajectory analyses contain errors that originate from factors including, but not limited to, the choice of input meteorological data, resolution of input data, and the vertical transport method used in trajectory calculations (Stohl et al., 1995; Cabello et al., 2008; Engström and Magnusson, 2009). Although the choice of meteorological data is the most important contributor to the uncertainties associated with trajectories calculations (Gebhart et al., 2005), no particular dataset has been found to be superior in terms of yielding the lowest error. While in this study we used GDAS data, which have been widely used as an input dataset for trajectory calculations even in regions with complicated topography (e.g., Tunved et al., 2013; Su et al., 2015), the aforementioned inherent errors should not be overlooked when interpreting the results presented in this work. Another factor that can contribute to the uncertainties for the results presented in this work is the use of GDAS as the source of precipitation data, as previous works (Sun et al., 2018; Nogueira, 2020) have demonstrated that there is some level of disagreement between precipitation datasets.

#### 3.1.1 Concentration-weighted trajectory analysis and seasonal rain maps

Concentration-weighted trajectories (CWTs) were calculated based on the 10 d back trajectories from HYSPLIT in conjunction with Bermuda surface PM_2.5_ data described in Sect. 2.1. The CWT method has been implemented widely to identify long-range pollutant transport pathways impacting a receptor site (Hsu et al., 2003; Wang et al., 2009; Hilario et al., 2020). Seasonal maps of average precipitation experienced by trajectories were also estimated based on 10 d back trajectories from HYSPLIT. The aforementioned analyses were performed for 0.5° × 0.5° grids covering the area encompassed by 10–80° N and 5–170° W. A weight function (*W_ij_* in Eq. 1) following the method of Dimitriou et al. (2015) was applied in the CWT analysis and precipitation maps to increase statistical stability. In Eq. (1), *n*_avg_ is the average number of trajectory endpoints per individual grid cell over the study region excluding cells with zero trajectory points, and *n_ij_* is the number of trajectory endpoints that lie in the grid cell (*i, j*).

(1) Wij={1nij>3navg0.71.5navg<nij<3navg0.4navg<nij<1.5navg0.2nij<navg}

10 d back trajectories were implemented for generating CWT and rain maps to illustrate potential distant sources impacting Bermuda. But for more quantitative analyses presented in the subsequent sections focused on transport most relevant to the WNAO region, 4 d back trajectories were used by simply truncating 10 d trajectories. The use of 4 d trajectories reduces the uncertainties associated with trajectory calculations in comparison to using 10 d trajectories and also enables us to focus on sources closer to the receptor site.

#### 3.1.2 Trajectory clustering

Hierarchical agglomerative clustering was used to identify characteristic trajectories reaching Bermuda at 100 m (a.g.l.). Hierarchical clustering was based on the “complete linkage” method (Govender and Sivakumar, 2020). 4 d HYSPLIT back trajectories were used to perform clustering analysis. Distances between trajectories were calculated using the Haversine formula, which calculates the distance between two points on Earth assuming they are on a great circle (Sinnott, 1984). The distance between any two trajectories was calculated as the sum of distances between trajectory end-points. Subsequently, clustering was conducted based on the symmetric distance matrix, which includes the distances between all pairs of trajectories. Clustering was performed for varying numbers of clusters ranging between 2 and 32. The L-method (Kassomenos et al., 2010) was implemented to identify the optimum number of clusters. In this method, root mean square deviation (RMSD) was calculated for each clustering run and then plotted versus the number of clusters to determine the optimum solution. RMSDs were estimated based on the distances between trajectories and associated mean cluster trajectories.

### 3.2 Airborne measurements

Airborne data from the Aerosol Cloud meteorology Interactions oVer the western ATlantic Experiment (ACTIVATE) are used from Research Flight 6 (RF6) on 22 February 2020. ACTIVATE involves two NASA Langley aircraft (HU-25 Falcon and UC-12 King Air) flying in coordination at different altitudes to simultaneously characterize the same vertical column with a focus on aerosol–cloud–meteorology interactions (Sorooshian et al., 2019). RF6 was a rare case of the HU-25 Falcon flying alone, but this aircraft conveniently included measurements relevant to this study. The ACTIVATE strategy involves the HU-25 Falcon flying in the boundary layer to characterize gas, aerosol, cloud, and meteorological parameters along the following level legs: min. alt. indicates the lowest altitude flown (~ 150 m), BCB is below cloud base, ACB is above cloud base, BCT is below cloud top, and ACT is above cloud top.

Data from the following instruments were used: condensation particle counter (CPC; TSI model 3772) for number concentration of particles with diameter > 10 nm; scanning mobility particle sizer (SMPS; TSI model 3081) for aerosol size distribution data between 3.2 and 89.1 nm; laser aerosol spectrometer (LAS; TSI model 3340) for aerosol size distribution data between diameters of 0.09 and 5 μm; two-dimensional optical array imaging probe (2DS; SPEC Inc.) (Lawson et al., 2006) for rain water content (RWC) quantified by integrating raindrop size distributions between diameters of 39.9 and 1464.9 μm; and fast cloud droplet probe (FCDP; SPEC Inc.) (Knop et al., 2021) for cloud liquid water content (LWC) calculated by integrating drop size distributions between diameters of 3 and 50 μm. With the exception of SMPS data (45 s resolution), all airborne data were at 1 s resolution.

### 3.3 Radionuclide tracers in the GEOS-Chem model

Lead-210 (^210^Pb, half-life 22.3 years) is the decay daughter of radon-222 (^222^Rn, half-life 3.8 d) emitted mainly from land surfaces. After production, it indiscriminately attaches to ambient submicron particles, which move with the air until being scavenged by precipitation or deposited to the surface. Because of its relatively well-known source and wet deposition as its principal sink, ^210^Pb has long been used to test wet deposition processes in global models (e.g., Liu et al., 2001). It is also a useful tracer to describe continental air influence over oceans. In this study, we use ^210^Pb as simulated by the GEOS-Chem model to investigate the role of precipitation scavenging in affecting seasonal surface aerosol concentrations at Bermuda.

GEOS-Chem (http://www.geos-chem.org, last access: 27 October 2021) is a global 3-D chemical transport model driven by meteorological fields from the Goddard Earth Observing System (GEOS) of the NASA Global Modeling and Assimilation Office (Bey et al., 2001; Eastham et al., 2014). It has been widely used to study trace gases and aerosols in the atmosphere. Here we use the model version 11-01 (http://wiki.seas.harvard.edu/geos-chem/index.php/GEOS-Chem_v11-01, last access: 27 October 2021) driven by the MERRA-2 reanalysis (at 2.5° longitude by 2° latitude resolution) to simulate ^222^Rn and ^210^Pb. The model simulates the emission, transport (advection, convection, boundary layer mixing), deposition, and decay of the radionuclide tracers (Liu et al., 2001; Brattich et al., 2017; Yu et al., 2018; Zhang et al., 2021). As a function of latitude, longitude, and month, ^222^Rn emission uses a customized emission scenario that was built upon previous estimates and evaluated against global ^222^Rn surface observations and vertical profile measurements (Zhang et al., 2021). GEOS-Chem uses the TPCORE advection algorithm of Lin and Rood (1996), calculates convective transport using archived convective mass fluxes (Wu et al., 2007), and uses the nonlocal boundary layer mixing scheme implemented by Lin and McElroy (2010). The wet deposition scheme follows that of Liu et al. (2001) and includes rainout (in-cloud scavenging) due to large-scale (stratiform and anvil) precipitation, scavenging in convective updrafts, and washout (below-cloud scavenging) by precipitation (Wang et al., 2011). A modification to the large-scale precipitation scavenging scheme is included to use spatiotemporally varying cloud water contents from MERRA-2 instead of a fixed constant value in the original model (Luo et al., 2019). Dry deposition is based on the resistance-in-series scheme of Wesely (1989).

## 4 Results and discussion

### 4.1 Seasonal profiles

#### 4.1.1 Back trajectories

Our results in Fig. 1 show that the summer months (June–August, JJA) are distinct due to the Bermuda High, promoting easterly winds at latitudes south of Bermuda that turn north and become southwesterly (approximately parallel to the US East Coast) towards Bermuda. The Bermuda high-pressure system and its associated anticyclonic circulation in the boundary layer have been reported to be strongest in April–September (Merrill, 1994; Moody et al., 1995). This high-pressure system breaks down in other months in favor of strengthened extratropical subpolar low pressure, thus yielding more air influence from the northwest and west (Arimoto et al., 1995; Davis et al., 1997), which is clearly evident in the other three seasonal panels in Fig. 1 and most pronounced in the winter months (December–February, DJF). In their analysis of air mass history leading to rain events over Bermuda, Altieri et al. (2013) observed more influence from air originating over water in warmer months (April–September) and faster-moving air masses originating over the continental US, primarily in the colder months of October–March. Moody and Galloway (1988) also showed that cool months (October–March) were marked by more transport from the US East Coast. It can be deduced from Fig. 1 that based on the farther-reaching source areas of the back trajectories in colder months, especially DJF, air moves faster in the boreal winter. Finally, we note that Figs. S1-S2 show the same results as Fig. 1 but with ending altitudes of 500 m and 1 km over Bermuda; the sensitivity tests indicate the same general results, and thus we continue the discussion using results based on 100 m.

#### 4.1.2 Surface aerosol and NO*_x_*

Recent work has shown a seasonal cycle over Bermuda for column-integrated aerosol properties, with aerosol optical depth (AOD) being highest in March–May (MAM) and JJA and lowest in September–November (SON) and DJF (Aldhaif et al., 2021). It was further shown that sea salt contributed more to AOD in the colder months (SON, DJF), whereas sulfate, organic carbon, black carbon, and dust were more dominant in MAM and JJA. In their examination of aerosol type seasonality at Bermuda, Huang et al. (1999) observed that marine and crustal elements peaked in winter and summer, respectively, and that pollution-derived particles dominated in spring with a smaller peak in fall. We use data from Fort Prospect station to gain a revised perspective about seasonality and the weekly cycle of surface layer aerosol and additionally NO*_x_* (box notch plots in Fig. S3a-f).

Median seasonal concentrations of PM_2.5_ (μgm^−3^) were as follows at Bermuda, being largely consistent with the AOD seasonal cycle: DJF = 5.50, MAM = 6.36, JJA = 6.11, SON = 5.33 (Fig. S3). NO*_x_* exhibited a similar seasonal pattern (ppbv): DJF = 17.76, MAM = 21.62, JJA = 18.68, SON = 13.95 (Fig. S3). It is difficult to ascertain sources and impacts of precipitation on PM_2.5_ based on these values. As a next step we present the seasonal CWT maps showing the predominant pathways accounting for the majority of PM_2.5_ at Bermuda (Fig. 2). Expectedly, PM_2.5_ in JJA is largely accounted for by trajectories following the general anticyclonic circulation already shown in Fig. 1c associated with the Bermuda High. These air masses are enriched with African dust as has been documented in many past studies (e.g., Arimoto, 2001; Huang et al., 1999; Muhs et al., 2012). In contrast, the other seasons (especially DJF and MAM) showed greater relative influence from North American outflow versus other source regions.

While we focus on long-range transport of PM_2.5_ to Bermuda, local sources cannot be ignored, including both sea salt and non-sea-salt species (e.g., Galloway et al., 1988). The island had a population of approximately 64 000 as of 2016 (Government of Bermuda, 2019). Local influence from anthropogenic sources has been reported to be insignificant in contrast to transported pollution (Galloway et al., 1988; Keene et al., 2014). We assess how significant local anthropogenic sources are based on day-of-week aerosol concentrations and whether significantly higher levels exist on working days compared to weekend days as shown in other regions with strong anthropogenic influence (Hilario et al., 2020, and references therein). Our analysis found negligible difference between working days (Monday–Friday) and weekend days (Saturday–Sunday) for both PM_2.5_ and NO*_x_* when analysis was done based on annual (Fig. S3b, d) or seasonal data (Figs. S4-S5). Therefore, it is less likely that local anthropogenic emissions dominate the island’s PM_2.5_ and NO*_x_*, providing support for transported sources being more influential; as will be shown, normalizing PM_2.5_ by CO helps control for local anthropogenic influence.

We also examined seasonal and day-of-week statistics for PM_10_ to assess the relative importance of coarse aerosol types including mainly sea salt and dust (Fig. S3e-f). Results reveal the highest median PM_10_ values (μgm^−3^) in DJF (19.24), followed by MAM (18.51), JJA (17.98), and SON (15.88). As will be shown later and already documented (Aldhaif et al., 2021), surface wind speeds around Bermuda are highest in DJF, contributing to higher sea salt emissions. Expectedly there was no observable PM_10_ weekly cycle as dust and sea salt are naturally emitted. Both PM_2.5_ and PM_10_ exhibited their highest seasonal standard deviations in JJA owing most likely to the episodic nature of some pollution events such as dust and biomass burning (e.g., Aldhaif et al., 2021).

#### 4.1.3 Precipitation along trajectories

Figure 3 shows seasonal profiles of average precipitation rate obtained from GDAS (Table 1) in 0.5° × 0.5° grids based on 10 d back trajectories arriving at Bermuda (100 m a.g.l.). The spatiotemporal pattern of precipitation over the WNAO is of the most interest in terms of potential impacts on wet scavenging of aerosol during the transport of North American pollution to Bermuda. In that regard, DJF shows the most pronounced levels of precipitation to the north and northwest of Bermuda over the WNAO, coincident with strong and frequent convection linked to frontogenesis (Painemal et al., 2021). This is consistent with how Painemal et al. (2021) showed that precipitation exhibits maximum levels over the Gulf Stream path owing to relatively high sea surface temperature and strong surface turbulent fluxes.

### 4.2 Trajectory clustering

**Figure 4.**
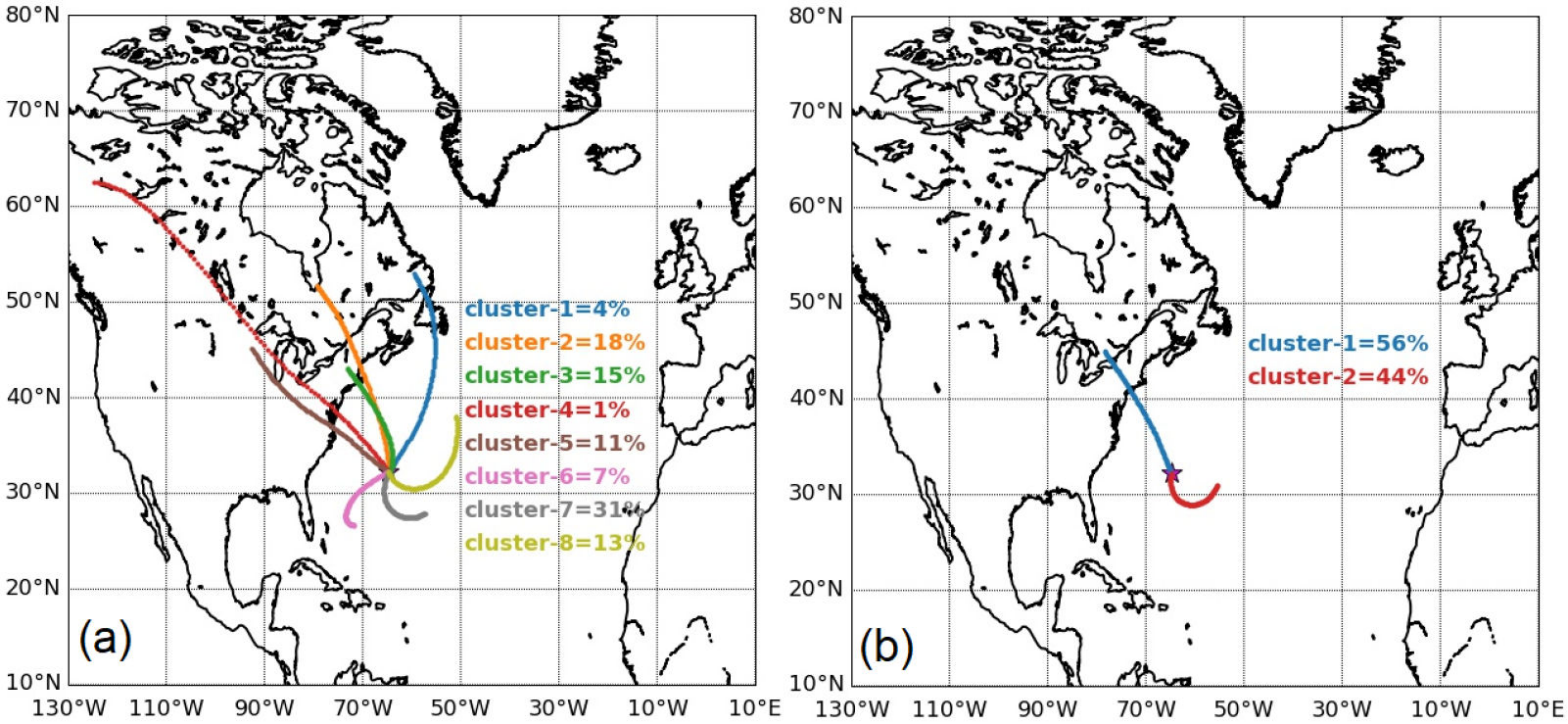
Cluster mean trajectories based on the **(a)** optimum solution having eight clusters and **(b)** a simplified solution with two clusters to enhance statistics for North American trajectories. Clustering was performed on 4 d HYSPLIT backward trajectories between 1 January 2015 and 31 December 2019.

Prior to examining how precipitation directly impacts PM_2.5_ at Bermuda, we identify characteristic trajectory pathways using the hierarchical agglomerative clustering method described in Sect. 2.3.2. We reiterate that this analysis is based on 4 d of back trajectories, rather than 10 d from Figs. 1-3, to focus more on transport closer to Bermuda. The optimum solution based on the L-method (see Sect. 2.3.2) resulted in eight trajectory clusters (Fig. 4a), with five (numbered 1–5) coming from North America and the remaining three (numbered 6–8) more characteristic of the anticyclonic circulation already described for JJA. The five former clusters account for 49 % of the total trajectories, with the latter three responsible for the remaining 51 %. The majority of trajectories from North America come offshore north of North Carolina (i.e., coastal areas north of ~ 35° N).

For the sake of simplicity of the remainder of the discussion, we reduced the number of characteristic trajectories to two (Fig. 4b) by conducting a new clustering analysis to have one from North America and the other from the southeast. Using only two clusters increases the number of data points in the North American cluster for more robust calculations of rain–aerosol relationships. Our choice to put together all North American air mass clusters in one group is aligned with a similar clustering choice by Chen and Duce (1983; see their Fig. 3) wherein trajectories were grouped together from Florida to the Canadian maritime provinces. Also, Mead et al. (2013) divided trajectory data ending at Bermuda into “Saharan” and “non-Saharan” seasons that generally coincide with our division of data into two clusters. Cluster 1 from North America accounts for 56 % of trajectories and Cluster 2 from the southeast is linked to 44 % of trajectories. It is clear from the two clusters that the North American air masses generally move faster as the characteristic 4 d back trajectories originate farther away from Bermuda than those of Cluster 2.

**Figure 5.**
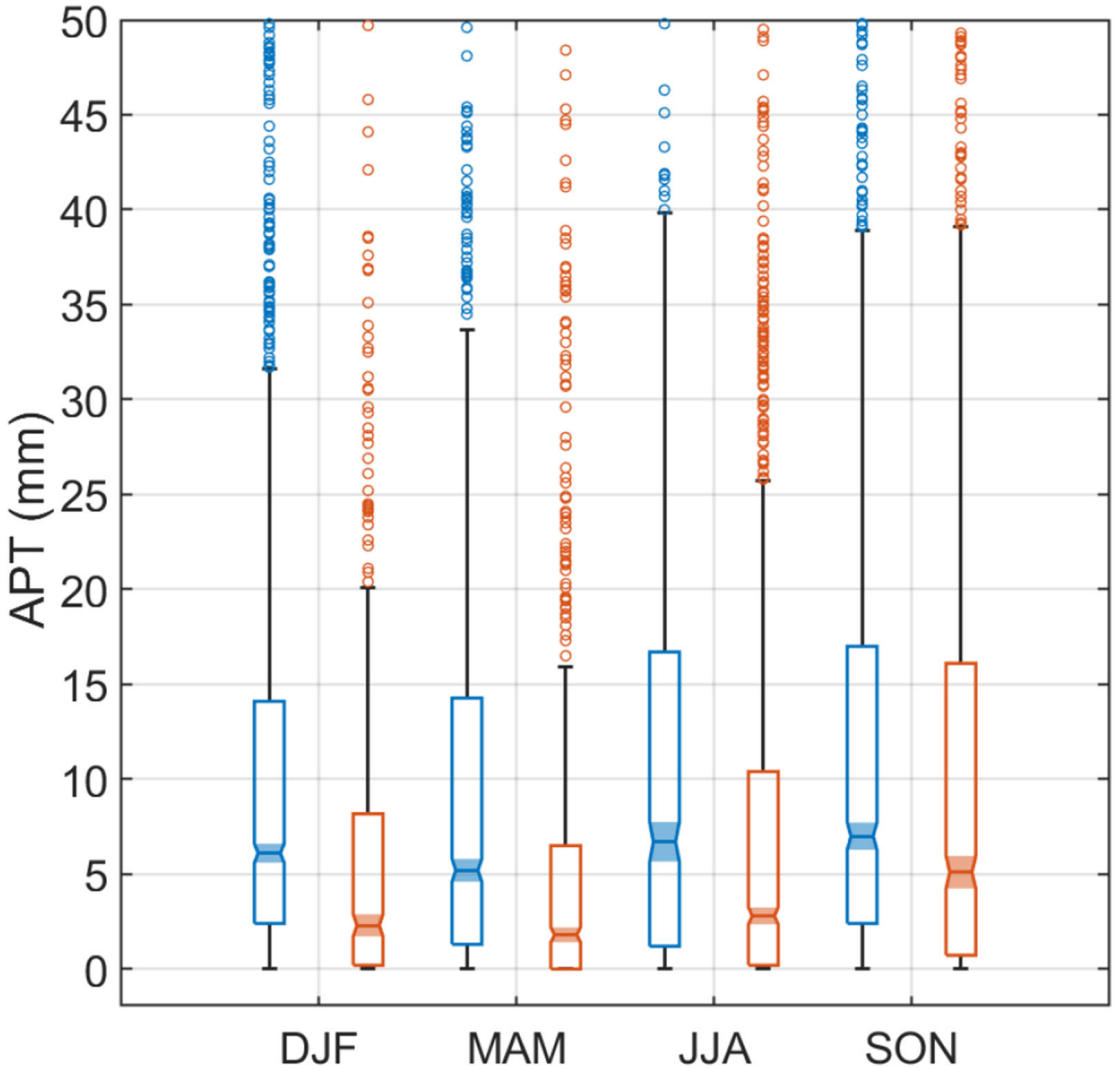
Box notch plot for each season comparing accumulated precipitation along trajectories (APT) for Clusters 1 (blue) and 2 (orange) from Fig. 4b. APT values were estimated from 4 d HYSPLIT back trajectories reaching Bermuda (32.30° N, 64.77° W) at 100 m a.g.l. The middle, bottom, and top lines in each box represent the median, 25th percentile, and 75th percentile, respectively. Markers show extreme values identified based on 1.5 × IQR (interquartile range) distance from the top of each box. Whiskers represent maximum and minimum values excluding extreme points. Boxes with notches and shaded regions that do not overlap have different medians at the 95 % confidence level.

Regardless of season, Cluster 1 was associated with higher APT values, with the seasonal median values (millimeters) as follows (Cluster1/Cluster 2): DJF = 6.1/2.3; MAM = 5.2/1.8; JJA = 6.7/2.8; SON = 7.0/5.1. Figure 5 shows a box notch plot comparing APT between clusters for each season, demonstrating statistically significant differences in median values between clusters for a given season at 95 % confidence. Furthermore, Cluster 1 exhibited higher CO levels at Bermuda for each season, with median values (units of ppbv) as follows (Cluster 1/Cluster 2): DJF = 89.7/76.3; MAM = 88.5/75.0; JJA = 68.9/58.7; SON = 81.6/65.6. Therefore, the combination of pollution outflow from North America and higher APT values makes Cluster 1 more relevant in terms of identifying potential wet scavenging effects on transported aerosol over the WNAO. The remainder of the study thus focuses on Cluster 1.

### 4.3 North America trajectory results

We next examine the relationship between APT and aerosol transport to Bermuda based on Cluster 1 results (Table 2). We compare data for “low” and “high” APT values based on thresholds for the 25th percentile (< 0.9 mm) and 75th percentile (> 13.5 mm), respectively, based on cumulative data from all seasons and years. As wet scavenging is expected to reduce PM_2.5_ during its transport from North America to Bermuda, we anticipate lower PM_2.5_ values at high APT. However, the results indicate this is only the case for MAM and JJA, with similar median values in SON and a higher median value in DJF for high-APT conditions. Interestingly, NO, NO_2_, NO*_x_*, and CO were all significantly higher in DJF for high-APT conditions too. This raises the issue that absolute PM_2.5_ concentrations should be normalized to account for the differences in concentration that existed closer to North America prior to potential wet scavenging over the WNAO.

To study the effects of wet removal processes on aerosol particles during long-range transport to a receptor site, many studies have used aerosol concentrations normalized by the concentration of an inert gaseous species co-emitted with particles at distance sources. Such normalization is critical and superior to the use of only aerosol concentration as the latter can be influenced by local sources that can mask aerosol response to removal processes during long-range transport. CO exhibits three important traits qualifying it as a species to normalize PM_2.5_ by: (i) being a reliable marker of anthropogenic pollution stemming from North America (Corral et al., 2021), (ii) being relatively insensitive to wet scavenging processes, and (iii) having a long lifetime in the atmosphere (~ 1 month; Weinstock, 1969) compared to aerosol particles. Consequently, we normalize PM_2.5_ by ΔCO to quantify transport efficiency and to reveal the potential effects of wet scavenging as has been done in past studies for other regions (Park et al., 2005; Garrett et al., 2010; Hilario et al., 2021; Matsui et al., 2011; Moteki et al., 2012; Oshima et al., 2012). We first determine the 5th percentile value of surface CO at Bermuda for each season for Cluster 1 trajectories and assume those are the seasonal background values as also done by Matsui et al. (2011). We then calculate ΔCO as the difference between each 6-hourly CO data point at Bermuda and the background value for a given season. We only use data when ΔCO > 3.2 ppbv to ensure a sufficiently high signal-to-noise ratio (Garrett et al., 2010).

**Figure 6.**
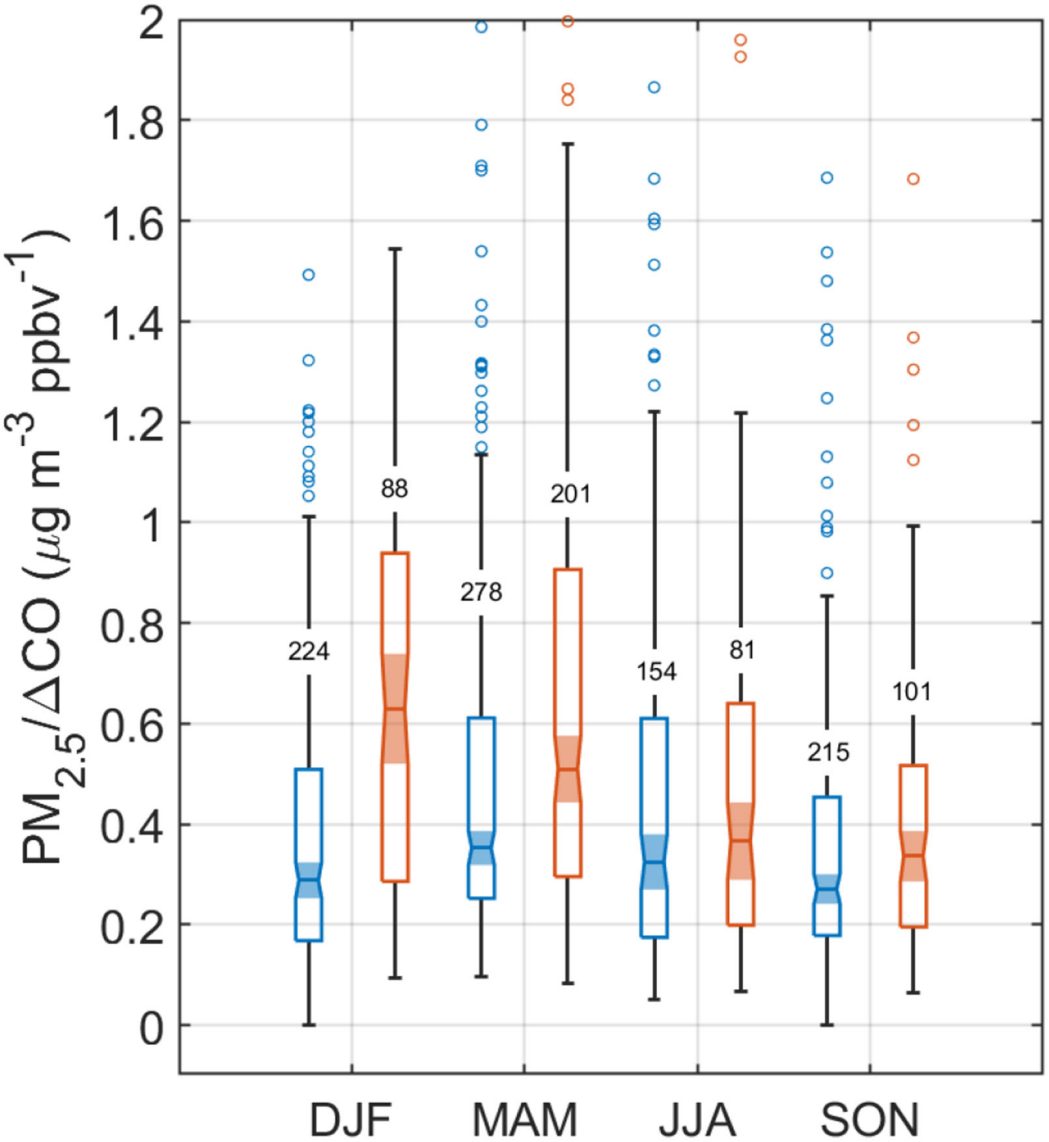
Box notch plot for each season comparing the PM_2.5_/ΔCO ratio for Cluster 1 trajectories for high-APT (blue) and low-APT (orange) conditions. APT thresholds are based on 25th (< 0.9 mm) and 75th (> 13.5 mm) percentiles of APT for all trajectories reaching Bermuda between 1 January 2015 and 31 December 2019. The number of samples in each group is placed on whiskers.

With the normalization technique, PM_2.5_/ΔCO exhibits lower values in the high-APT category for each season compared to low-APT conditions (Fig. 6), with differences between medians being statistically significant in DJF and MAM based on *p* value < 0.05 with a Wilcoxon rank-sum test (Table 2). The DJF season exhibits the greatest reduction of this ratio (by 53 %) in high-APT conditions (0.29 μgm^−3^ ppbv^−1^ versus 0.62 μgm^−3^ ppbv^−1^ based on median values; Table 2). Therefore, these results suggest that it is plausible that wet scavenging has a marked impact on surface PM_2.5_ at a remote ocean site in the WNAO. This also helps support the speculation proposed by Aldhaif et al. (2021) that wet scavenging can reconcile why, in particular for DJF, the high density of trajectories coming from North America correlates with a reduction in fine particulate pollution arriving at Bermuda compared to other seasons. It is noteworthy that the highest median value of PM_2.5_/ΔCO was for the low-APT category of DJF, providing support for how that season has both a greater influence of aerosol transport from North America (when the precipitation scavenging potential is reduced during low-APT periods) and the greatest sensitivity to the effects of precipitation over the WNAO owing to the widest range in this ratio’s value between high- and low-APT categories.

**Figure 7.**
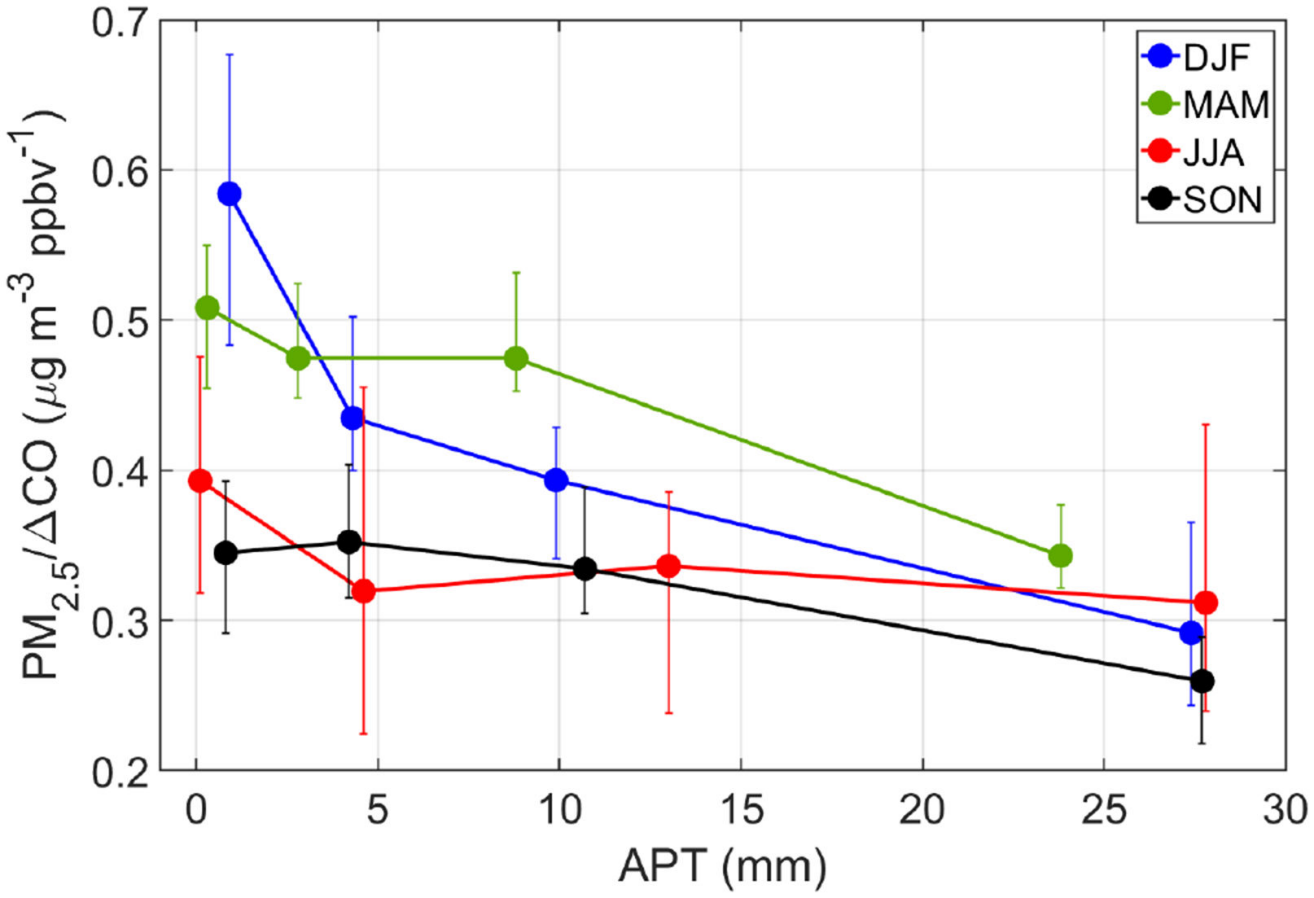
Seasonal sensitivity of PM_2.5_/ΔCO to APT for Cluster 1 trajectories, divided based on four APT bins that have a similar number of data points per season. Markers denote median values, and error bars represent the 95 % confidence interval for medians based on a bootstrapping method (*n* = 100 000). Number of points per marker: DJF = 192–194; MAM = 247–251; JJA = 107–110; SON = 183–191.

Figure 7 additionally shows the seasonal sensitivity of PM_2.5_/ΔCO to APT based on four bins of APT (bin ranges shown in Table S3) chosen in such a way to provide similar numbers of data points per bin for each particular season. We note that the general trends are preserved using similar bin ranges in each of the seasons. DJF and MAM show the greatest reductions from the first to last bin as expected based on Table 2, but these were also the only two seasons showing reductions between each successive bin. In contrast, SON and JJA exhibited more variable behavior, with PM_2.5_/ΔCO actually increasing between a pair of bins in each season. A number of reasons can potentially explain the less pronounced reduction in PM_2.5_/ΔCO for SON and JJA: (i) lower values to begin with in the lowest APT bins (and thus lower potential for scavenging to occur), (ii) potential humidity effects associated with air masses at higher APT values promoting secondary aerosol formation (Huang et al., 2014; Quan et al., 2015; Ding et al., 2021), and (iii) more influence from natural emissions in the form of dust (especially JJA) and sea salt (especially SON) (Aldhaif et al., 2021). Another noteworthy result is that the season with the clearest scavenging signature (DJF) shows the most sensitivity (i.e., steepest downward slope) between the first two APT bins (0.9 mm versus 4.3 mm) as there was a 26 % reduction in PM_2.5_/ΔCO (0.584 to 0.435 μg m^−3^ ppbv^−1^), resulting in a slope (units of μgm^−3^ ppbv^−1^ mm^−1^) of −0.044 in contrast to slopes of −0.007 and −0.006 for the two subsequent pairs of bins in DJF. Tunved et al. (2013) also reported a similar exponential trend between particle mass and accumulated precipitation, with an initial rapid decrease in particle mass followed by a decreased removal rate of aerosol due to precipitation.

We next address some additional details motivated by values shown in Table 2. We examine three aerosol constituents linked to anthropogenic outflow from North America, including sulfate, black carbon (BC), and organic carbon (OC), from MERRA-2 reanalysis. We recognize that sulfate and OC have non-anthropogenic precursor vapors such as ocean-emitted dimethyl sulfide and biogenic volatile organic compounds, respectively. Being the most abundant of the three, sulfate exhibits the same characteristics as PM_2.5_ when normalized by ΔCO, with the sharpest reduction at high-APT conditions in DJF, followed by MAM, and then finally by JJA and SON albeit with *p* values > 0.05 for the latter two seasons compared to low-APT conditions. BC / ΔCO ratios show the same relative characteristics between APT categories as sulfate / ΔCO for each season and mostly the same for OC / ΔCO except that the reduction in the median value in high-APT conditions for SON was significant (*p* value < 0.05). Regardless of season, but most pronounced in DJF, was the consistent result that OC/ΔCO exhibited the highest relative reduction at high-APT conditions (versus low-APT conditions) compared to BC and sulfate. Further work with more expansive observational data is needed to better understand how different species respond to wet scavenging.

Normalization by ΔCO was important for assessing transport efficiency of anthropogenic pollution, but we also considered dust and sea salt without ΔCO normalization as they are predominantly emitted by natural sources. Although outside the scope of this study, we caution that MERRA-2 concentrations of sea salt in the PM_2.5_ fraction may exceed those of total PM_2.5_ as measured at Fort Prospect (Table 2) owing to the inherent differences in the two respective datasets including the larger spatial scale covered by MERRA-2 compared to the point measurements at Fort Prospect. Previous analysis of precipitation scavenging ratios over Bermuda showed that larger aerosol types (e.g., sea salt) are removed more efficiently than smaller aerosol types (e.g., sulfate, nitrate) (Galloway et al., 1993). Total sea salt and sea salt in the PM_2.5_ fraction exhibited higher median concentrations for the high-APT category (*p* value < 0.05) for all seasons except JJA, which had more comparable values. This can be explained by how days experiencing high APT exhibited significantly higher surface wind speeds around Bermuda for all seasons except JJA, for which wind speeds in general were depressed. Therefore, the reduction of the PM_2.5_/ΔCO ratio in high-APT conditions may actually be an underestimate of wet scavenging of North American pollution outflow since local sea salt is higher on windier days marked by high APT.

To put this last assertion on firmer ground, we examined local rain values as they could be influential in terms of scavenging the locally generated sea salt. The median values of local rain on high-APT days for each season based on APT for the most recent 6 h of trajectories arriving at Bermuda (APT_6 h_) were 0.0–0.1 mm, while median values of APT_6 h_ on low-APT days were 0 mm in each season. The only significant difference in median APT_6 h_ values was in DJF when it was 0.1 mm on high-APT days in contrast to 0.0 mm on low-APT days. Therefore, for DJF the slightly enhanced APT_6 h_ can possibly offset the greater sea salt emissions in terms of impacting PM_2.5_ levels over Bermuda. Results for the other major natural aerosol type (dust) reveal much lower overall concentrations compared to sea salt for both bulk sizes and the PM_2.5_ fraction. There was no consistent trend across the four seasons in terms of dust levels being higher for either the low- or high-APT category, which is not unexpected as dust is not a major aerosol type expected from North American outflow (Yu et al., 2020; Corral et al., 2021).

#### 4.3.1 Volume size distributions

**Figure 8.**
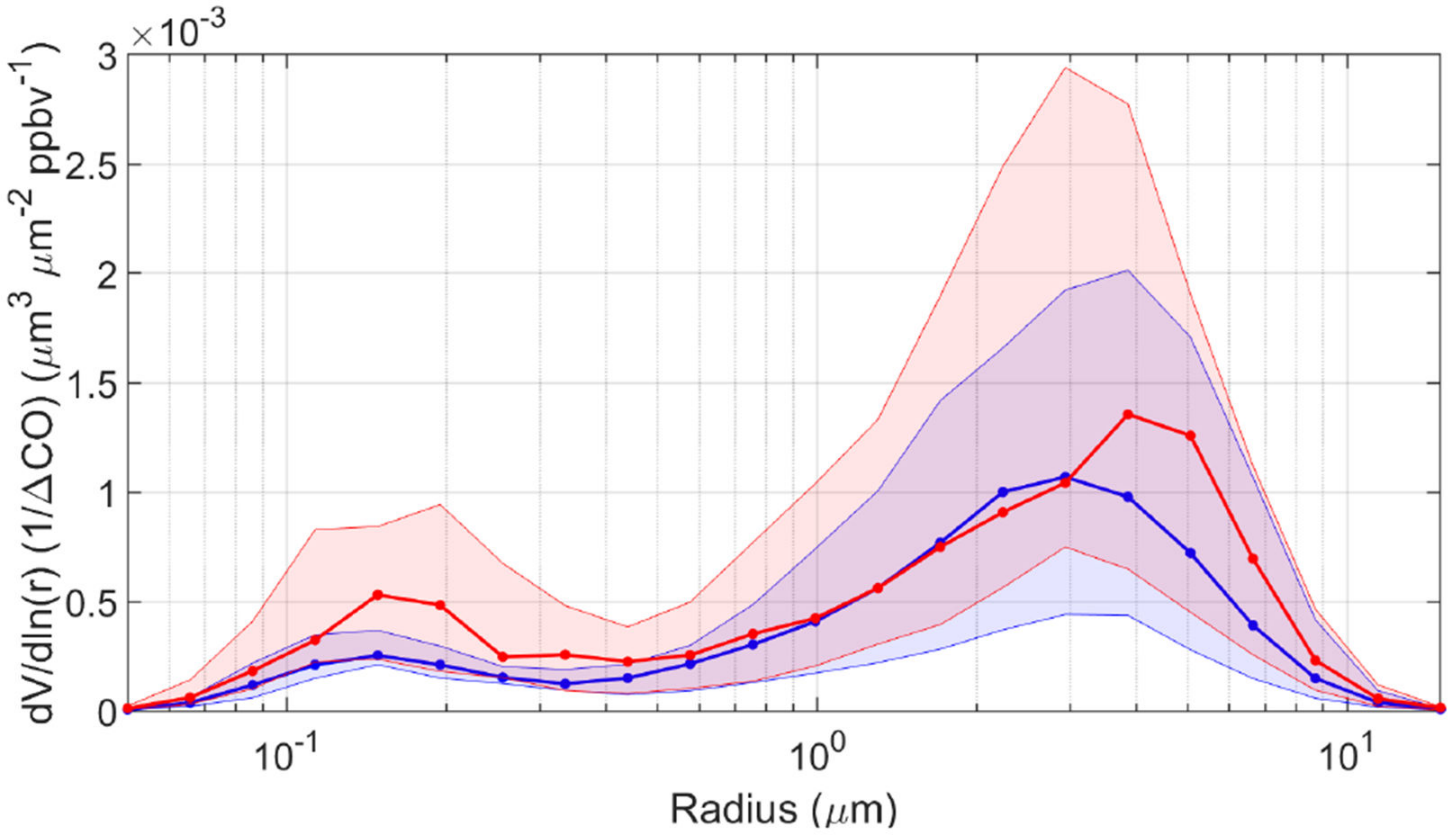
Volume size distributions (VSDs) normalized by ΔCO for high-APT (> 13 mm; blue, *n* = 16) and low-APT (< 0.9 mm; red, n *=* 19) groups for Cluster 1 trajectories. Thick curves correspond to medians, and shaded areas extend to the 25th and 75th percentiles. VSDs are based on AERONET data between 1 January 2015 and 31 December 2019.

We next examine AERONET volume size distribution (VSD) relationships with APT. We normalize the volume concentration data by corresponding ΔCO in the same way as was done for PM_2.5_, with the same condition of using data only when ΔCO > 3.2ppbv. A few cautionary details are first noted about these data in comparison to APT: (i) there are limited VSD data in the AERONET dataset, which is why we use all seasons of data together for Fig. 8 and Table 2; (ii) AERONET data are representative of ambient conditions, and changes in relative humidity can influence VSD profiles; and (iii) AERONET data are column-based and not necessarily representative of only the surface layer where the trajectories end in our analysis of HYSPLIT data. Related to the last point, past work noted that column optical properties over Bermuda can be weakly correlated with such measurements at the surface (Aryal et al., 2014) due largely to aerosol layers aloft (Ennis and Sievering, 1990). At the same time, studies have shown that there can be enhanced number and volume concentrations in the marine boundary layer versus the free troposphere over Bermuda (Horvath et al., 1990; Kim et al., 1990).

The median VSDs for both APT categories exhibit a bimodal profile with a more dominant coarse mode, consistent with what is already known for Bermuda based on AERONET data (Aldhaif et al., 2021). The unique aspect of this work is that in high-APT conditions, there is a reduction in median volume concentration in the smaller mode between radii of 0.05 and ~ 1 μm, with a slight enhancement on the leading shoulder of the larger mode between radii of 1.71 and 2.94 μm (Fig. 8). The greatest relative reductions in the fine mode, which is more indicative of transported continental pollution, occurred between midpoint radii of 0.15 and 0.33 μm with relative reductions in those four bins (i.e., midpoint radii 0.15, 0.19, 0.26, and 0.33 μm) ranging from 38 % to 52 %. The coarse mode peaked at larger radii (3.86 μm) in low-APT conditions relative to high-APT conditions (2.94 μm).

Table 2 reports VSD parameter values for the APT categories separated by fine and coarse modes. Although only significantly different based on 90% confidence (*p* value = 0.09), the fine-mode volume concentration normalized by ΔCO in the high-APT category was less than half (45 %) the value in the low-APT category. There were insignificant differences between effective radii and volume median radii, in addition to the geometric standard deviation for the fine mode between APT categories. For the coarse mode, only the geometric standard deviation exhibited a significant difference by being higher in the high-APT category (0.684 versus 0.647), although we presume that has less to do with actual scavenging effects and more to do with different times of the year when the relative abundance of different coarse particle type changes.

The AERONET results support the idea that scavenging on high-APT days efficiently removes fine particulate matter but that there can still be appreciable levels of locally generated sea salt due to higher local surface winds on high-APT days. Related to the columnar nature of AERONET data, it is important to note that others have reported large-scale subsidence of pollution from the middle and upper troposphere, especially in spring, based on enhanced ozone mixing ratios at the surface of Bermuda (Oltmans and Levy, 1992; Cooper et al., 1998; Milne et al., 2000; Li et al., 2002). Moreover, this phenomenon is synoptically favorable with the transport of North American polluted air behind cold fronts, especially in spring (Moody et al., 1995), and often linked to the lifting of polluted air out of the boundary layer by convection over the continental US (Prados et al., 1999). It is unclear based on the current dataset how effective these events were in impacting either the surface layer or columnar-based aerosol measurements at Bermuda.

### 4.4 GEOS-Chem model results

We conduct four GEOS-Chem simulations of the ^210^Pb submicron aerosol tracer including (a) one standard simulation, (b) the same as the standard simulation but with the ^222^Rn tracer emissions from the North American continent (25–60° N, 130–70° W) removed, (c) the same as the standard simulation but without ^210^Pb scavenging due to large-scale precipitation, and (d) the same as the standard simulation but without ^210^Pb scavenging by convective precipitation. The difference between (a) and (b) quantifies the North American contribution to atmospheric ^210^Pb concentrations. The difference between (a) and (c) reflects the role of large-scale precipitation scavenging, while the difference between (a) and (d) reflects that of convective precipitation scavenging in determining atmospheric ^210^Pb concentrations. All model simulations are conducted for the period from September 2016 to December 2017 with the first 4 months for spin-up. Monthly mean outputs for 2017 are used for analysis, which is a representative year within the time frame of the analysis presented in Sect. 3.1-3.3. This is confirmed by the seasonal APT box chart constructed in Fig. S6 using only 2017 data, which nearly follows the trend observed when the 5-year data are used (Fig. 5).

**Figure 9.**
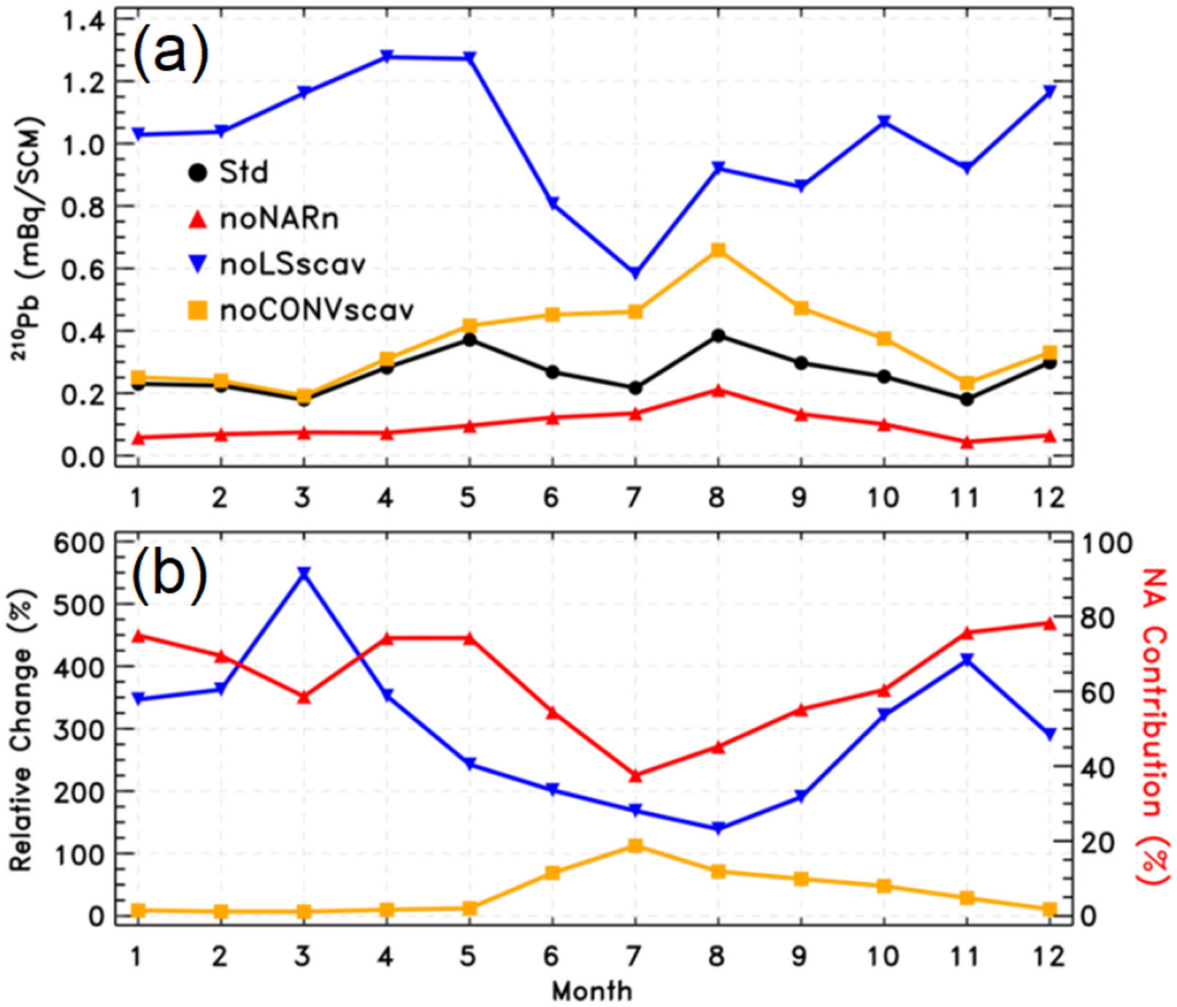
Simulated monthly surface ^210^Pb tracer concentrations of submicron milliBecquerels (mBq) per standard cubic meter of (dry) air (mBqSCM^−1^) at Bermuda (32.31° N, 64.75° W) in 2017 as a way to assess effects of precipitation scavenging on North American outflow. Panel **(a)** shows monthly mean surface ^210^Pb concentrations in the standard simulation (Std) and three sensitivity simulations, i.e., without North American ^222^Rn emissions (noNARn), without large-scale precipitation scavenging (noLSscav), and without convective precipitation scavenging (noCONVscav). Panel **(b)** shows percentage changes, i.e., (noLSscav-Std)/Std × 100 in blue and (noCONVscav-Std)/Std × 100 in orange, and the North American contribution in red, i.e., (Std-noNARn)/Std × 100.

Figure 9a shows monthly mean surface ^210^Pb concentrations at Bermuda for 2017 in the standard simulation and three sensitivity simulations. Figure 9b plots the relative changes in simulated ^210^Pb concentrations due to the effects of large-scale or convective precipitation scavenging. Also included in Fig. 9b is the North American contribution. The standard model simulates a seasonality in ^210^Pb concentrations with two distinct peaks in May and August (upper panel). The May peak is a result of increased transport from North America in combination with reduced scavenging. In contrast, the August peak results from long-range transport from other continents (e.g., North Africa, Europe) along the southern edge of the Bermuda High. The lows in March and November are attributed to strong large-scale precipitation scavenging, and the low in July is associated with enhanced convective precipitation scavenging. The sensitivity simulations clearly show that the role of large-scale precipitation scavenging in affecting surface ^210^Pb concentrations at Bermuda is much larger in winter and spring than in summer, with a maximum in March (lower panel), while convective scavenging also plays an important role in summer. The relative contribution of North American ^222^Rn emissions is largest in winter (~ 75 %−80 %), suggesting air masses reaching Bermuda often experience large-scale precipitation scavenging while traveling from the North American continent during winter. While the model may have limitations and inherent uncertainties, its results are at least consistent with results already shown, putting our conclusions on firmer ground.

### 4.5 Airborne case study

**Figure 10.**
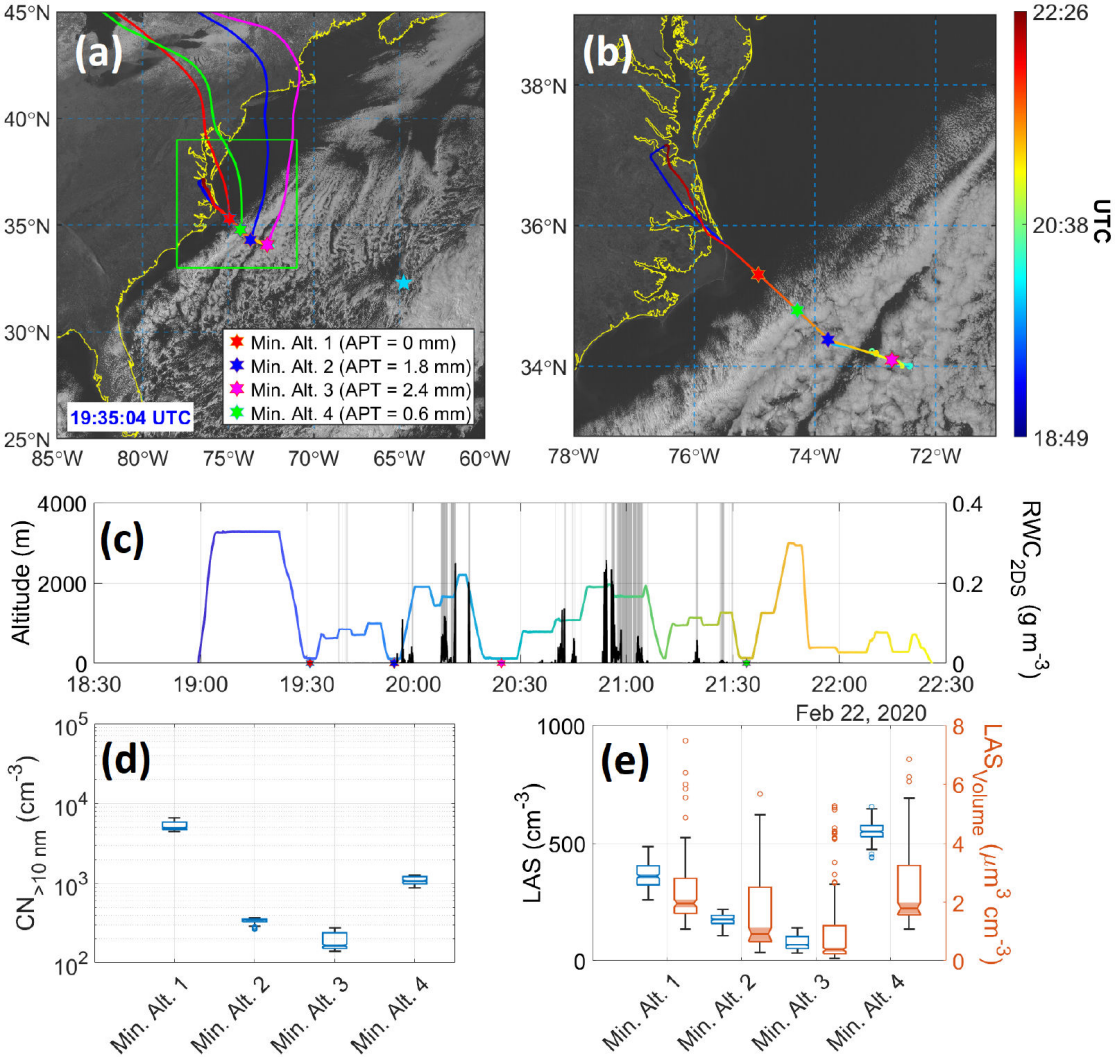
Summary of ACTIVATE’s Research Flight 6 on 22 February 2020. **(a)** HU-25 Falcon flight track overlaid on GOES-16 imagery of the WNAO (Bermuda denoted by blue star) also showing 96 h back trajectories calculated for each respective min. alt. leg. The midpoints of the four min. alt. legs are marked, including values for the accumulated precipitation along the trajectory (APT) for the recent history of the sampled air masses when they were over the ocean (time over land excluded from APT calculation). **(b)** Zoomed-in version of panel **(a)** focused on the flight path. **(c)** Time series of Falcon altitude colored by flight UTC time (color bar in panel **b**) and rain water content (RWC) from the 2DS probe. Gray shaded bars signify when FCDP liquid water content exceeded 0.05 gm^−3^, indicative of cloud legs. The same four colored stars from **(a)** are shown on the *x* axis to indicate where they occurred. **(d–e)** Box notch plots of the leg-mean min. alt. values of CPC particle concentration (> 0.01 μm) and the number and volume concentrations of the LAS (> 0.09 μm).

The DJF season has been shown in this study to exhibit the greatest potential for wet scavenging and the highest density of trajectories from North America reaching Bermuda. To probe deeper now, we take advantage of data from ACTIVATE RF6 on 22 February 2020, which characterized the intermediate region between North America and Bermuda. Weather in the ACTIVATE domain on this day was characterized by a transition from post-cold-front conditions to high pressure. A cold front passed over Bermuda the previous day at approximately 18:00 UTC on 21 February and by the flight period of RF06 was approximately 600 km southeast of the island. Meanwhile, a broad but weakening area of surface high pressure continued eastward into the southeast US. Winds in the boundary layer were southwesterly at around 5ms^−1^ near the base of operations (NASA Langley Research Center; Hampton, Virginia), which were associated with a weak trough on the northeast side of the high-pressure system. These winds shifted to north-northwest near the coast at 2.5 ms^−1^ and north-northeast at 7.4 ms^−1^ near the far end of the flight track; Bermuda reported north-northeast winds around 9ms^−1^ during this period. Aloft, 500 hPa flow was from the west-northwest. NASA Langley reported few to no clouds during the flight period, while Bermuda reported broken clouds with multiple layers (with bases around 900 and 1800 m) and rain showers at or near the airport. This is consistent with satellite imagery (Fig. 10a), which shows an area of scattered to broken cumulus and stratocumulus extending from the cold front near Bermuda to the edge of the Gulf Stream off the US East Coast. Satellite-retrieved cloud bases were at 1–2 km, with cloud tops ranging from 1.5–3.5 km; from the HU-25 Falcon flight legs, cloud bases encountered along the flight track were 750–1100 m and cloud tops were 1200–1800 m.

Figure 10a shows the general flight path, which involved flying to a point southeast of the operations base (Hampton, Virginia) and then retracing the path back to land. Four HYSPLIT back trajectories are shown (Fig. 10a), corresponding to midpoints of each min. alt. leg when the aircraft was at its lowest altitude (~ 150 m). APT calculations were conducted for segments of those four trajectories that were over the ocean to focus on wet removal clouds over the WNAO. Negligible rain accumulated up to the point of the min. alt. 1 leg, as there were cloud-free conditions between land and that offshore point. In contrast, the next three min. alt. legs show higher APT values ranging from 0.6 to 2.4 mm, consistent with the GOES-16 imagery showing cloud fraction increasing just to the southeast of the min. alt. 1 leg. Expectedly, APT values progressively increased with offshore distance as a result of air masses being exposed to clouds for longer periods. Figure S7 shows 27 trajectories obtained for each min. alt. leg based on ensemble trajectory analysis, which is a technique available in HYSPLIT to evaluate uncertainties in trajectory calculations by offsetting the meteorological data by a fixed grid factor. Average APT values based on ensemble analysis (Fig. S7) were 0.29, 1.18, 2.27, and 0.73 mm, corresponding to min. alt. 1, 2, 3, and 4 legs, respectively, which follow the trend observed in Fig. 10.

**Figure 11.**
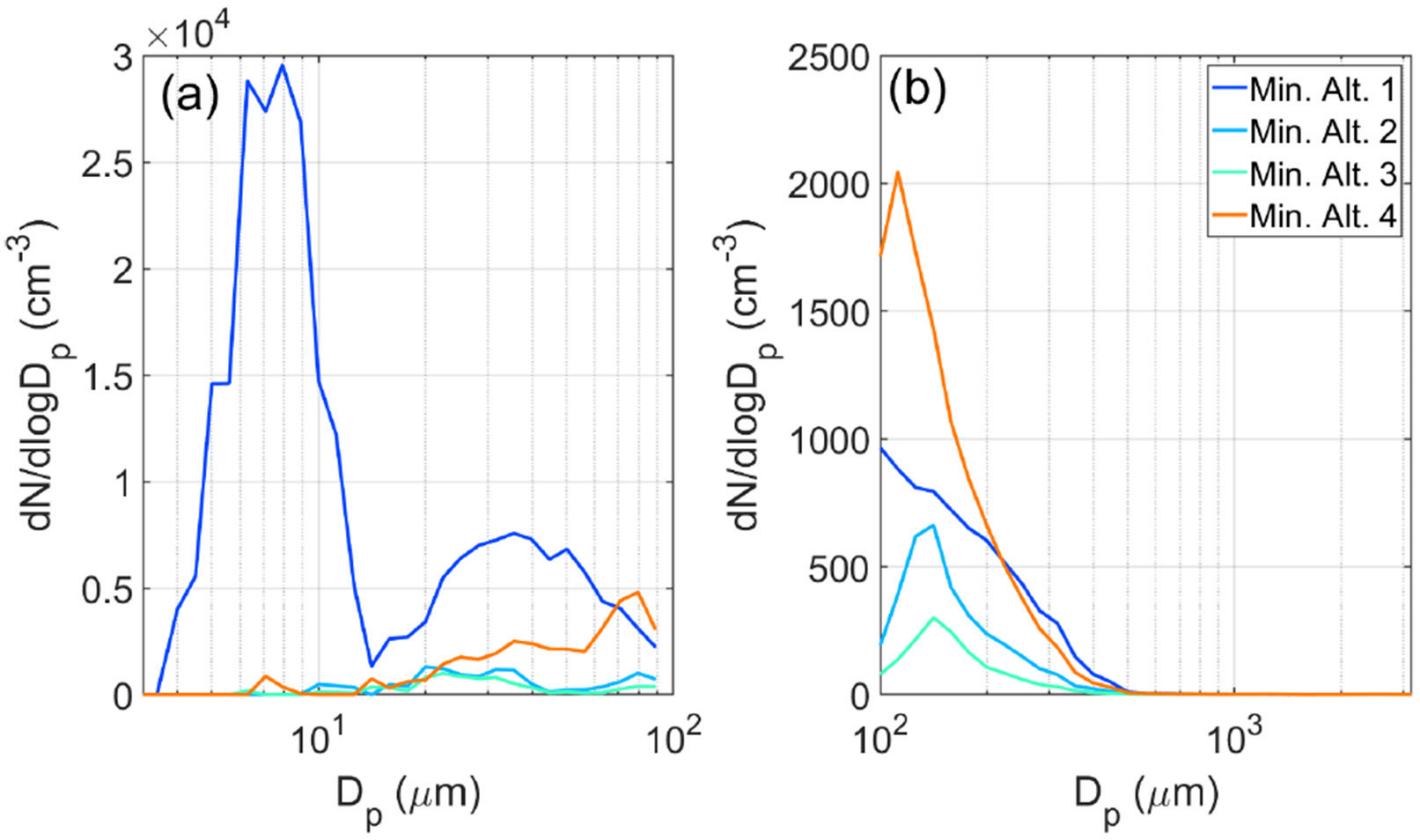
Aerosol size distribution comparison (**a** SMPS and **b** LAS) between the four HU-25 Falcon min. alt. legs during ACTIVATE Research Flight 6, as shown in Fig. 10.

Shortly after the min. alt. 1 leg, the Falcon conducted two consecutive pairs of BCB and ACB legs (i.e., below cloud base followed by above cloud base), followed by a slant descent to the min. alt. 2 leg, where RWC values were enhanced (up to 0.02 g m^−3^ at 19:55:22UTC) owing to precipitation from overlying clouds. Very shortly thereafter, RWC reached as high as 0.11 g m^−3^ (19:56:50 UTC) in the slant ascent profile passing through clouds. The APT value in the min. alt. 2 leg was 1.8 mm. A significant reduction was observed in the aerosol number and volume concentrations for the min. alt. 2 leg compared to the min. alt. 1 leg (Fig. 10d-e). Table S4 reports the statistics for aerosol parameters measured in min. alt. legs (Fig. 10). CPC (> 10 nm) concentrations dropped by 93 % from a leg median value of 4938 cm^−3^ during min. alt. 1 to 345 cm^−3^ during min. alt. 2, whereas the LAS number and volume (> 100 nm) concentrations dropped from 360 to 174 cm^−3^ and from 2.0 to 0.9 μm^3^ cm^−3^, respectively. Size distribution data in those two legs show a significant reduction in particle concentration across the full diameter range as measured by the SMPS and LAS (Fig. 11). A notable feature from the SMPS was a pronounced peak between 3.5 and 14.1 nm, suggestive of nucleation, that was absent in subsequent min. alt. legs, presumably owing to some combination of coagulation and scavenging.

The aircraft continued southeast after the min. alt. 2 leg and passed through more patches of precipitation, leading to the highest APT value of 2.4 mm in the min. alt. 3 leg, for which leg median values were as follows: CPC 165 cm^−3^, LAS number 66 cm^−3^, LAS volume 0.4 μm^3^ cm^−3^. While the SMPS distributions in the min. alt. 2 and 3 legs were very similar, the LAS size distribution in the min. alt. 3 leg is shifted towards lower concentrations, especially below 400 nm. On the path back towards Virginia, the Falcon conducted one final min. alt. 4 leg right before the boundary between cloudy and clear air, with the APT value being 0.6 mm. Between the min. alt. 3 and 4 legs, significant RWC values were again observed, reaching as high as 0.26 gm^−3^ at 20:54:20 UTC. Aerosol concentration measurements increased relative to the min. alt. 2 and 3 legs (leg median values): CPC = 1076 cm^−3^, LAS number = 545 cm^−3^, LAS volume = 1.8 μm^3^ cm^−3^. It is difficult to compare results from the min. alt. 1 and 4 legs as ~ 2 h had passed and there were different conditions impacting the two respective sampled air masses. The size distributions varied considerably for the min. alt. 4 leg compared to the other three legs, with increased concentrations between 20 and 200 nm, presumably as a result of continued pollution outflow and more photochemistry and aerosol growth processing compared to earlier in the day.

To conclude, it is plausible based on the case flight data that the emerging presence of clouds and precipitation led to the substantial reduction of aerosol particles with distance offshore via wet scavenging processes. Further research is warranted with more extensive data to move closer to showing causal relationships between precipitation and aerosol particles. For instance, a few points of caution from RF6 are worth mentioning. First, the coastal trajectories in Fig. 10 corresponding to the different Min Alt. legs originated from varying places extending from the Virginia coast up north towards Cape Cod, Massachusetts. Secondly, cloud dynamics and boundary layer structure can vary offshore. Related to the latter, PBLH data obtained from MERRA-2 along the flight track revealed that there were deeper boundary layers farther offshore, but not sufficiently deeper to fully explain the reductions in aerosol concentration: PBLH corresponding to min. alt. legs 1, 2, 3, and 4 of 1156, 1728, 1740, and 1530 m, respectively. Lastly, aerosol concentrations linked to continental outflow naturally decrease offshore anyway, including in cloud-free conditions, owing to dilution during transport.

## 5 Conclusions

This study examines the sensitivity of surface aerosol characteristics over a remote area of the western North Atlantic Ocean (Bermuda) to precipitation along trajectories coming from North America. Based on trajectory clustering with HYSPLIT data, two characteristic transport corridors to Bermuda’s surface layer (100ma.g.l.) were identified, with the focus being the one coming from North America (Cluster 1). Seasonal analysis of HYSPLIT and Bermuda surface data showed that JJA is distinct in terms of having transport from the southeast with the other seasons, especially DJF, having more North American influence with higher concentrations of CO. Comparing Cluster 1 trajectory data between high (> 13.5 mm) and low (< 0.9 mm) accumulated precipitation along trajectories (APT), there was a clear signature of wet scavenging effects by precipitation with more than a 2-fold reduction in PM_2.5_/ΔCO in DJF (0.29 μgm^−3^ ppbv^−1^ versus 0.62 μgm^−3^ ppbv^−1^), with the reduction being less severe for other seasons. The greatest sensitivity of PM_2.5_/ΔCO to APT was at the lowest values (up to ~5mm; slope of −0.044 μgm^−3^ ppbv^−1^ mm^−1^), above which the descending slope of PM_2.5_/ΔCO versus APT was less steep.

Speciated data indicate that anthropogenic species such as sulfate, black carbon, and organic carbon are reduced as a function of APT (much like PM_2.5_). However, sea salt was not necessarily reduced and at times could even be higher at Bermuda with high-APT conditions, which is attributed to higher local wind speeds. Analysis of AERONET volume size distribution data at Bermuda confirms the substantial reduction of fine-mode volume concentrations in contrast to a smaller change in the coarse mode on high-APT days. GEOS-Chem simulations of the radionuclide aerosol tracer ^210^Pb confirm that North American influence at the surface of Bermuda is highest in DJF, with those air masses significantly impacted by large-scale (i.e., stratiform and anvil) precipitation scavenging; furthermore, convective scavenging is shown to play an important role in summer months. A research flight from ACTIVATE on 22 February 2020 demonstrates a significant gradient in aerosol number and volume concentrations offshore of North America as soon as trajectories start passing across clouds, consistent with increasing APT away from the coast leading to increased aerosol particle removal.

Our results have implications for other remote marine regions impacted by transport of continental emissions. These results also highlight the important role of precipitation in modifying aerosol levels, potentially including their vertical distribution (e.g., Luan and Jaeglé, 2013), along continental outflow trajectories. We show that cloud and precipitation processes along trajectories have significant impacts on resultant aerosol characteristics. Therefore, it is important to strongly constrain wet scavenging processes in models to improve aerosol forecasting over the WNAO.

## Supplementary Material

Supplement

## Figures and Tables

**Table 1.** Summary of datasets used in this work. Data are between 1 January 2015 and 31 December 2019, with the exception of ACTIVATE aircraft data based on a single flight day on 22 February 2020. Section 2 provides more details about the datasets used in this study, including specific instruments from the ACTIVATE airborne dataset.

Parameter	Acronym	Data source	Spatial resolution	Time resolution
Particulate matter mass concentration (aerodynamic diameter less than 2.5 μm)	PM_2.5_	Fort Prospect station	–	Hourly
Particulate matter mass concentration (aerodynamic diameter less than 10 μm)	PM_10_	Fort Prospect station	–	Daily
Nitrogen monoxide concentration	NO	Fort Prospect station	–	Hourly
Nitrogen dioxide concentration	NO_2_	Fort Prospect station	–	Hourly
Nitrogen oxide concentration	NO*_x_*	Fort Prospect station	–	Hourly
Volume size distribution	VSD	AERONET	–	Hourly
Carbon monoxide surface concentration	CO	MERRA-2	0.625° (long.) × 0.5° (lat.)	Hourly
Aerosol speciated surface mass concentrations	–	MERRA-2	0.625° (long.) × 0.5° (lat.)	Hourly
Surface wind speed	Wind_SF_	MERRA-2	0.625° (long.) × 0.5° (lat.)	Hourly
Planetary boundary layer height	PBLH	MERRA-2	0.625° (long.) × 0.5° (lat.)	Hourly
Precipitation	APT (rain)	GDAS	1° × 1°	Hourly
Aerosol–cloud properties	–	Airborne: ACTIVATE	–	1–45 s

**Table 2.** Seasonal medians of aerosol, gas, and meteorological variables for Cluster 1 divided into high- and low-APT categories. Differences in median values that are statistically significant (*p* value < 0.05) based on a Wilcoxon rank-sum test are highlighted with italic font. Percentage differences* between high- and low-APT median values are provided in parentheses. NO, NO_2_, NO*_x_*, and PM_2.5_ are based on Fort Prospect measurements, whereas all other parameters are from MERRA-2 with the exception of the two APT rows (derived from HYSPLIT and GDAS) and the last eight rows corresponding to AERONET volume size distribution data. We combined all seasons for AERONET data to have sufficient statistics for comparisons (high APT 16 points, low APT 19 points). AERONET parameters include volume concentration (*V*), effective radii (*R*_eff_), volume median radii (*R*), and geometric standard deviation (*σ*) with subscripts f and c for fine and coarse modes, respectively. The number of data points for each table entry is summarized in Table S2.

	High-rain (APT > 13.5 mm) / low-rain (APT < 0.9 mm) (% difference*)			
Parameter	DJF	MAM	JJA	SON
NO (ppbv)	*6.0/3.5 (71 %)*	7.3/7.8(−6 %)	*8.3/13.1 (−37 %)*	3.8/4.2 (−10 %)
NO_2_ (ppbv)	*13.9/12.8 (9 %)*	13.4/12.0 (12 %)	*8.6/6.6 (30 %)*	9.4/9.2 (2 %)
NO*_x_* (ppbv)	*19.6/17.5 (12 %)*	21.2/21.8 (−3 %)	*17.4/23.3 (−25 %)*	14.1/14.2 (−1 %)
CO (ppbv)	*97.8/84.7 (15 %)*	*92.4/88.6 (4 %)*	*70.8/65.9 (7 %)*	83.7/81.4 (3 %)
PM_2.5_ (μgm^−3^)	*6.1/5.5 (11 %)*	*6.7/7.3 (−8 %)*	*5.9/7.8 (−24 %)*	5.5/5.1 (8 %)
PM_2.5_/ΔCO (μgm^−3^ ppbv^−1^)	*0.29/0.62 (−53 %)*	*0.35/0.51 (−31 %)*	0.32/0.37 (−14 %)	0.27/0.33 (−18 %)
Sea salt (μgm^−3^)	*47.2/28.4 (66 %)*	*44.1/25.4 (74 %)*	27.0/26.0 (4 %)	*50.6/36.0 (41 %)*
Sea salt_PM2.5_ (μgm^−3^)	*6.2/4.0 (55 %)*	*6.2/4.1 (51 %)*	4.9/4.9 (0 %)	*6.8/5.0 (36 %)*
Dust (μgm^−3^)	*0.80/0.91 (−12 %)*	*2.32/3.03 (−23 %)*	*4.47/3.02 (48 %)*	*1.16/1.04 (12 %)*
Dust_PM2.5_ (μgm^−3^)	*0.31/0.34 (−9 %)*	*0.79/1.00 (−21 %)*	*1.58/1.18 (34 %)*	*0.44/0.36 (22 %)*
Sea salt/ΔCO (μgm^−3^ ppbv^−1^)	*2.10/2.74 (−23 %)*	*2.54/1.70 (49 %)*	1.50/1.58 (−5 %)	*2.44/1.66 (47 %)*
Sulfate/ΔCO (μgm^−3^ ppbv^−1^)	*0.029/0.055 (−47 %)*	*0.041/0.052 (−21 %)*	0.039/0.046 (−15 %)	0.024/0.027 (−11 %)
Dust/ΔCO (μgm^−3^ ppbv^−1^)	*0.038/0.082 (−54 %)*	*0.129/0.186 (−31 %)*	*0.235/0.152 (55 %)*	0.052/0.047 (11 %)
BC/μCO (μg m^−3^ ppbv^−1^)	*0.0031/0.0056 (−45 %)*	*0.0042/0.0057 (−26 %)*	0.0041/0.0049 (−16 %)	0.0032/0.0033 (−3 %)
OC/ΔCO (μg m^−3^ ppbv^−1^)	*0.0093/0.0238 (−61 %)*	*0.0164/0.0276 (−41 %)*	0.0225/0.0287 (−22 %)	*0.0127/0.0153 (−17 %)*
Sea salt_PM2.5_/ΔCO (μgm^−3^ ppbv^−1^)	*0.263/0.403 (−35 %)*	*0.352/0.262 (34 %)*	0.284/0.298 (−5 %)	*0.331/0.255 (30 %)*
Dust_PM2.5_/ΔCO (μgm^−3^ ppbv^−1^)	*0.015/0.033 (−55 %)*	*0.042/0.062 (−32 %)*	*0.087/0.053 (64 %)*	0.018/0.017 (6 %)
Wind_SF_ (ms^−1^)	*8.5/7.1 (20 %)*	*8.4/5.9 (42 %)*	4.4/4.7 (−6 %)	*7.7/6.6 (17 %)*
APT_6h_ (mm)	*0.1/0.0 (NaN)*	0.0/0.0 (NaN)	0.0/0.0 (NaN)	0.0/0.0 (NaN)
APT (mm)	*24.7/0.0 (NaN)*	*22.6/0.2 (11200 %)*	*24.1/0.0 (NaN)*	*25.0/0.2 (12400 %)*
	All			
*V*_f_/ΔCO × 10^4^ (μm^3^ μm^−2^ ppbv^−1^)	3.42/7.55 (−55 %)			
*R*_eff-f_ (μm)	0.158/0.147 (7 %)			
*R*_f_ (μm)	0.176/0.171 (3 %)			
*σ*_f_	0.471/0.470 (0 %)			
*V*_c_/ΔCO × 10^4^ (μm^3^ μm^−2^ ppbv^−1^)	2.04/2.12 (−4 %)			
*R*_eff-c_ (μm)	1.956/2.085 (−6 %)			
*R*_c_ (μm)	2.503/2.562 (−2 %)			
*σ*_c_	*0.684/0.647 (6 %)*			

* $\%\;\text{difference}=\frac{X_{\text{high-rain}}-X_{\text{low-rain}}}{X_{\text{low-rain}}}\times 100$

## Data Availability

Fort Prospect station aerosol and gas measurements can be found at https://doi.org/10.6084/m9.figshare.13651454.v2 (Peters, 2021). AERONET data can be found at https://aeronet.gsfc.nasa.gov/(AERONET, 2020). HYSPLIT data as described by Stein et al. (2015) can be found at https://www.ready.noaa.gov/HYSPLIT.php. MERRA-2 data can be found at https://disc.gsfc.nasa.gov/ (Global Modeling and Assimilation Office (GMAO), 2021). The GEOS-Chem model as described by Bey et al. (2001) can be found at http://wiki.seas.harvard.edu/geos-chem/index.php/GEOS-Chem_v11-01. ACTIVATE Airborne Data can be found at https://doi.org/10.5067/ASDC/ACTIVATE_Aerosol_AircraftInSitu_Falcon_Data_1 (NASA/LARC/SD/ASDC, 2020a), https://doi.org/10.5067/ASDC/ACTIVATE_Cloud_AircraftInSitu_Falcon_Data_1 (NASA/LARC/SD/ASDC, 2020b), and https://doi.org/10.5067/ASDC/ACTIVATE_MetNav_AircraftInSitu_Falcon_Data_1 (NASA/LARC/SD/ASDC, 2020c). Section 3.5 airport weather data can be found at http://mesonet.agron.iastate.edu/ASOS/ (Iowa State University of Science and Technology, 2021). Section 3.5 ocean surface analysis charts and GFS 500 hPa analysis data can be found at https://www.ncei.noaa.gov/data/ncep-charts/access/ (National Centers for Environmental Information (NCEI), 2021a). Section 3.5 North America analysis-satellite composite can be found at https://www.wpc.ncep.noaa.gov/archives/web_pages/sfc/sfc_archive_maps.php (National Centers for Environmental Information (NCEI), 2021b). Section 3.5 satellite imagery and products can be found at https://satcorps.larc.nasa.gov/cgi-bin/site/showdoc?docid=4&cmd=field-experiment-homepage&exp=ACTIVATE. (NASA/Langley SatCORPS, 2021).
