# Supplementary material for: Aerosol responses to precipitation along North American air trajectories arriving at Bermuda
Source: Atmos Chem Phys. Author manuscript; Available in PMC 2021 Nov 23. (PMC8609468; doi:10.5194/acp-21-16121-2021)
Supplement: Supplement [file NIHMS1754081-supplement-Supplement.pdf]

Supplement of Atmos. Chem. Phys., 21, 16121–16141, 2021
https://doi.org/10.5194/acp-21-16121-2021-supplement
© Author(s) 2021. CC BY 4.0 License.

Atmospheric Chemistry and Physics
Open Access

Supplement of

Aerosol responses to precipitation along North American air trajectories arriving at Bermuda

Hossein Dadashazar et al.

Correspondence to: Hossein Dadashazar (hosseind@arizona.edu)

The copyright of individual parts of the supplement might differ from the article licence.

Table S1. Seasonal number of points available for various measurements conducted at Fort Prospect in Bermuda between Jan 2015 and Dec 2019. It should be noted that data points correspond to 6-hour resolution for all variables except for PM_{10} data that were at daily resolution.

	DJF	MAM	JJA	SON
NO	1340	1626	1698	1758
NO_2	1340	1626	1698	1758
NO_X	1340	1626	1698	1758
$PM_{2.5}$	1295	1761	1712	1551
PM_{10}	57	66	72	59

Table S2. Number of points that were used to calculate statistics presented in Table 2.

	High-rain (APT > 13.5 mm)/Low-rain (APT < 0.9 mm)			
Parameter	DJF	MAM	JJA	SON
NO (ppbv)	232/147	252/206	166/112	290/141
NO_2 (ppbv)	232/147	252/206	166/112	290/141
NO_x (ppbv)	232/147	252/206	166/112	290/141
CO (ppbv)	356/171	308/244	173/120	307/148
$PM_{2.5}$ (μg m^{-3})	244/132	290/242	168/111	255/117
$PM_{2.5}$/ΔCO (μg m^{-3} $ppbv^{-1}$)	224/87	278/209	154/78	215/98
Sea-Salt (μg m^{-3})	356/171	308/244	173/120	307/148
$\text{Sea-Salt}_{PM2.5}$ (μg m^{-3})	356/171	308/244	173/120	307/148
Dust (μg m^{-3})	356/171	308/244	173/120	307/148
$\text{Dust}_{PM2.5}$ (μg m^{-3})	356/171	308/244	173/120	307/148
Sea-Salt/ΔCO (μg m^{-3} $ppbv^{-1}$)	327/124	294/210	157/85	259/124
Sulfate/ΔCO (μg m^{-3} $ppbv^{-1}$)	327/124	294/210	157/85	259/124
Dust/ΔCO (μg m^{-3} $ppbv^{-1}$)	327/124	294/210	157/85	259/124
BC/ΔCO (μg m^{-3} $ppbv^{-1}$)	327/124	294/210	157/85	259/124
OC/ΔCO (μg m^{-3} $ppbv^{-1}$)	327/124	294/210	157/85	259/124
$\text{Sea-Salt}_{PM2.5}$/ΔCO (μg m^{-3} $ppbv^{-1}$)	327/124	294/210	157/85	259/124
$\text{Dust}_{PM2.5}$/ΔCO (μg m^{-3} $ppbv^{-1}$)	327/124	294/210	157/85	259/124
Wind_{SF} (m s^{-1})	356/171	308/244	173/120	307/148
APT_{6h} (mm)	356/171	308/244	173/120	307/148
APT (mm)	356/171	308/244	173/120	307/148
	All			
V_f /ΔCO × 10^4 ($μm^3$ $μm^{-2}$ $ppbv^{-1}$)	16/19			
R_{eff-f} (μm)	16/19			
R_f (μm)	16/19			
$σ_f$	16/19			
V_c /ΔCO × 10^4 ($μm^3$ $μm^{-2}$ $ppbv^{-1}$)	16/19			
R_{eff-c} (μm)	16/19			
R_c (μm)	16/19			
$σ_c$	16/19			

Table S3. Four APT bin ranges (mm) that were used to create seasonal plots shown in Fig. 7.

Bin Number	DJF	MAM	JJA	SON
1	0-2.5	0-1.4	0-1.8	0-2.4
2	2.5-6.7	1.4-5.3	1.8-8.7	2.4-7.2
3	6.7-15.7	5.3-14.6	8.7-19.0	7.2-16.0
4	15.7-164.9	14.6-118.9	19.0-74.2	16.0-106.2

Table S4. Median values of aerosol parameters and APT for Min. Alt. legs (Fig. 10) conducted in ACTIVATE's Research Flight 6 on 22 February 2020.

Parameter	Min. Alt. 1	Min. Alt. 2	Min. Alt. 3	Min. Alt. 4
APT (mm)	0.0	1.8	2.4	0.6
$CN_{>10nm}$ (cm^{-3})	4938	345	165	1076
LAS (cm^{-3})	360	174	66	550
LAS_{Volume} (μm^3 cm^{-3})	2.0	0.9	0.4	1.8

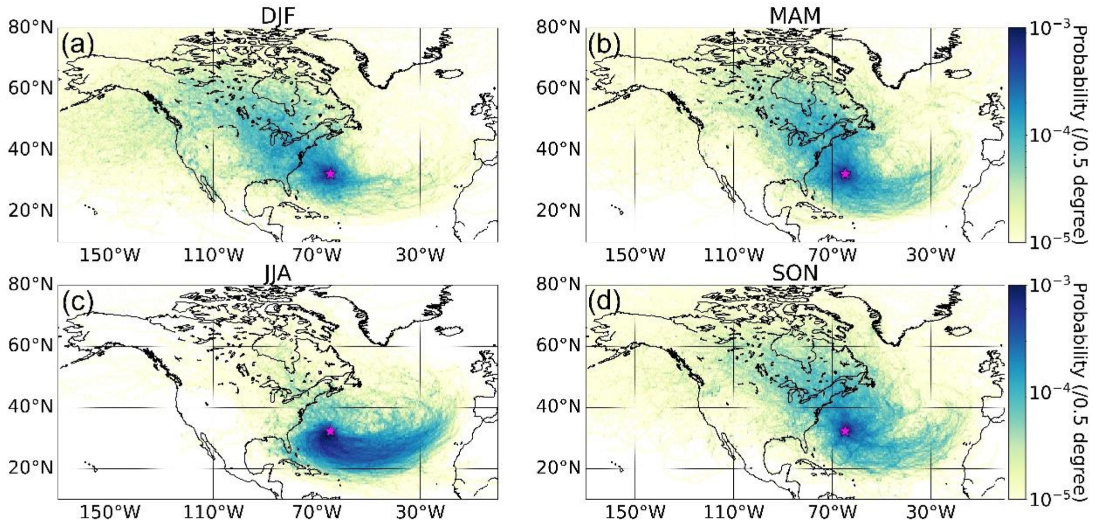

Figure S1. Seasonal maps (a-d) showing the probability density of trajectories calculated based on 10-day HYSPLIT backward trajectories reaching Bermuda (32.30° N, 64.77°W), denoted by the pink star, at 500 m (AGL). This analysis is based on trajectories between 01 January 2015 and 31 December 2019.

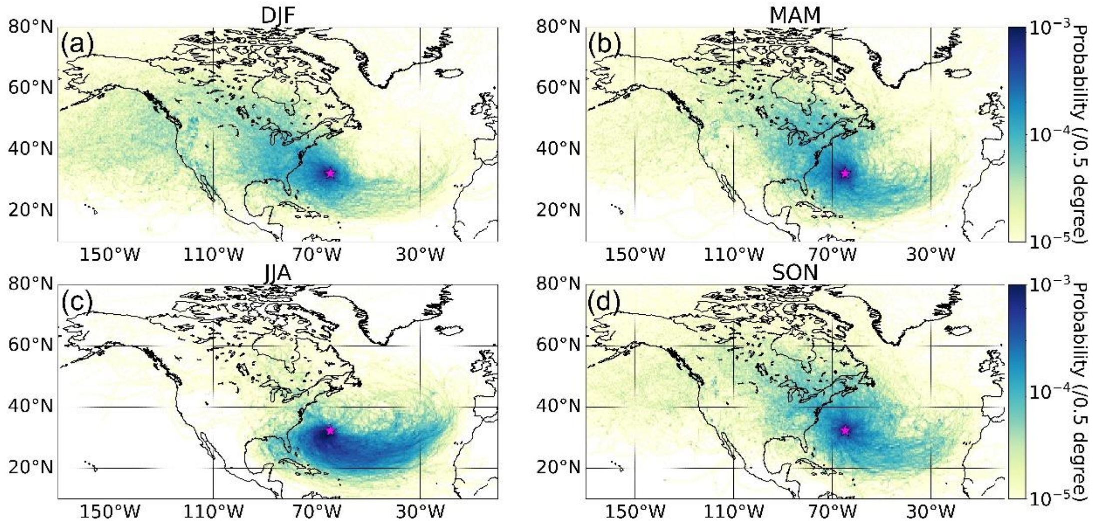

Figure S2. Seasonal maps (a-d) showing the probability density of trajectories calculated based on 10-day HYSPLIT backward trajectories reaching Bermuda (32.30° N, 64.77°W), denoted by the pink star, at 1 km (AGL). This analysis is based on trajectories between 01 January 2015 and 31 December 2019.

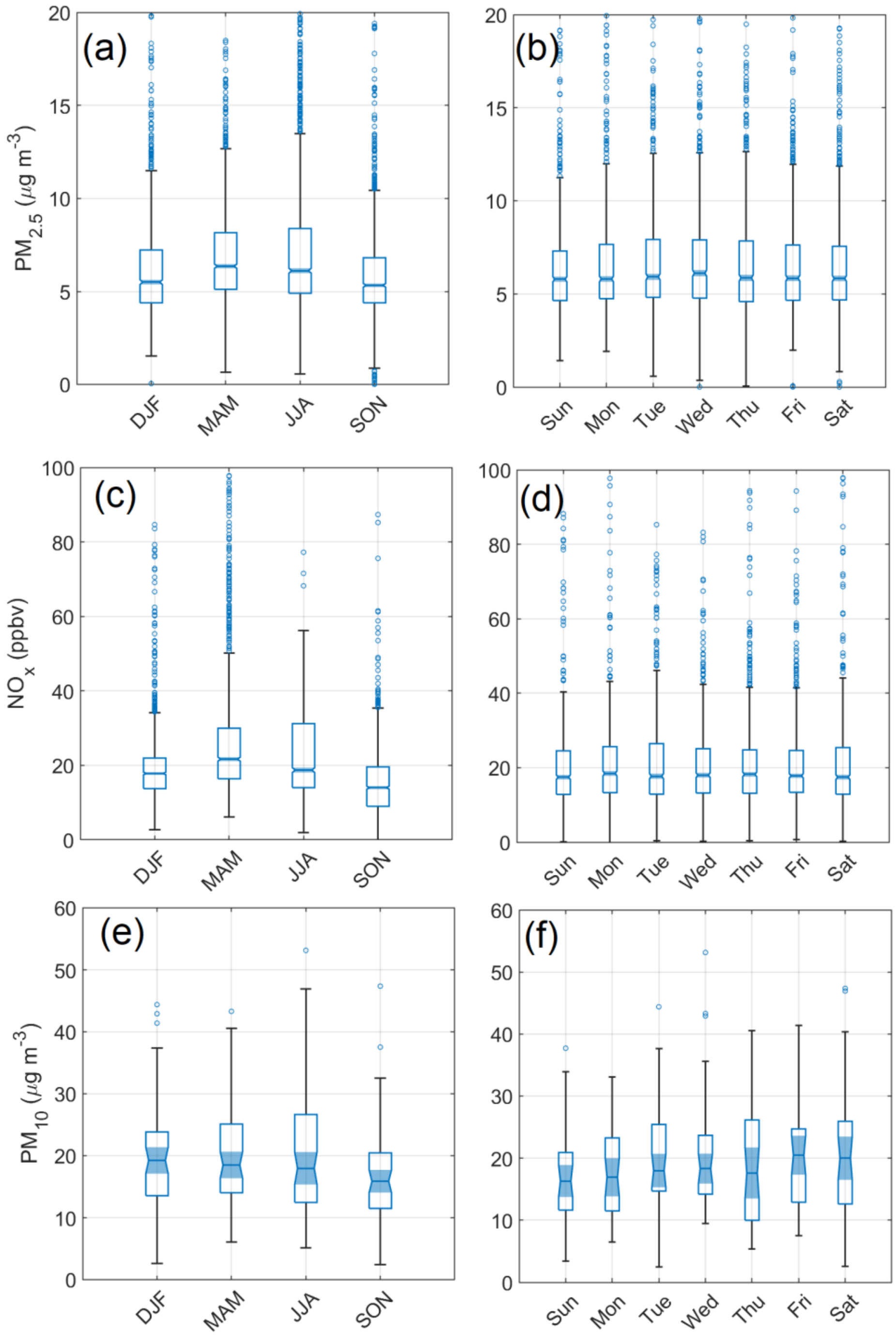

Figure S3. Seasonal (panels a/c/e) and day-of-week (panels b/d/f) box notch plots of $PM_{2.5}$, NO_x, and PM_{10} measured at Fort Prospect in Bermuda between 1 January 2015 and 31 December 2019. The middle, bottom, and top lines in each box represent the median, 25th percentile, and 75th percentile, respectively. Markers show extreme values identified based on 1.5×IQR (interquartile range) distance from the top and bottom of each box. Whiskers represent maximum and minimum values excluding extreme points. Boxes with notches and shaded regions that do not overlap have different medians at 95% confidence level.

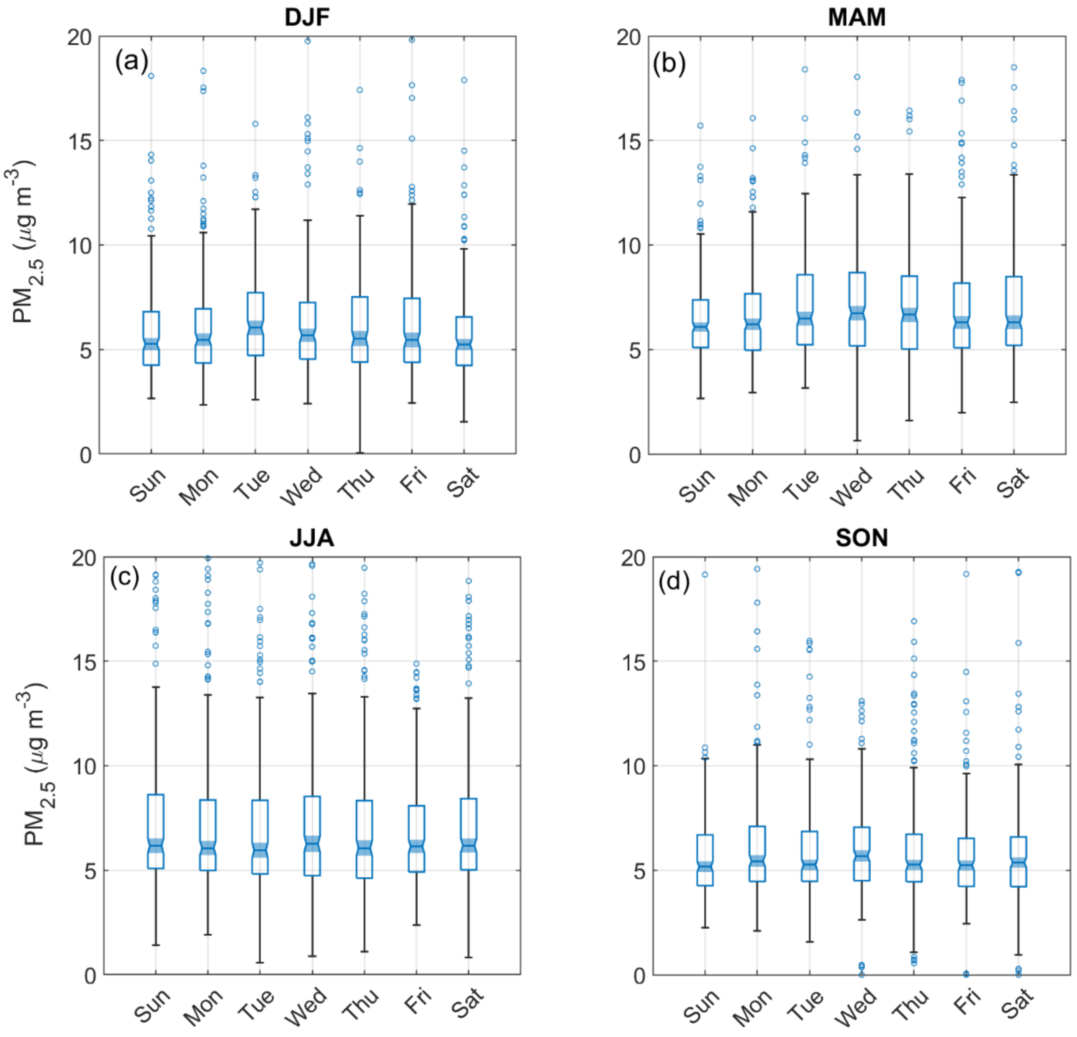

Figure S4. Day-of-week box notch plots of $PM_{2.5}$ for (a-d) different seasons measured at Fort Prospect in Bermuda between 1 January 2015 and 31 December 2019.

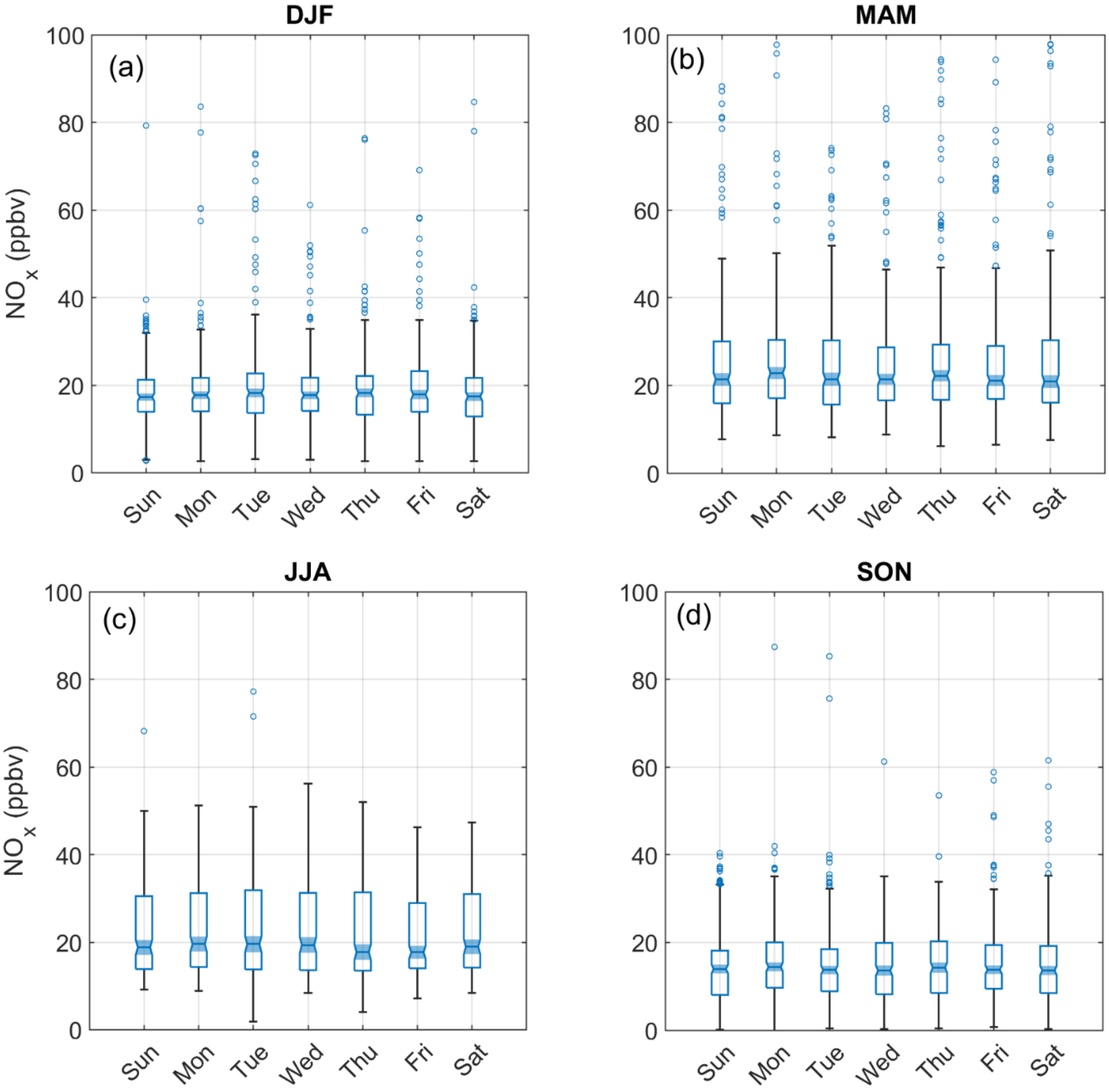

Figure S5. Day-of-week box notch plots of NO$_x$ for (a-d) different seasons measured at Fort Prospect in Bermuda between 1 January 2015 and 31 December 2019.

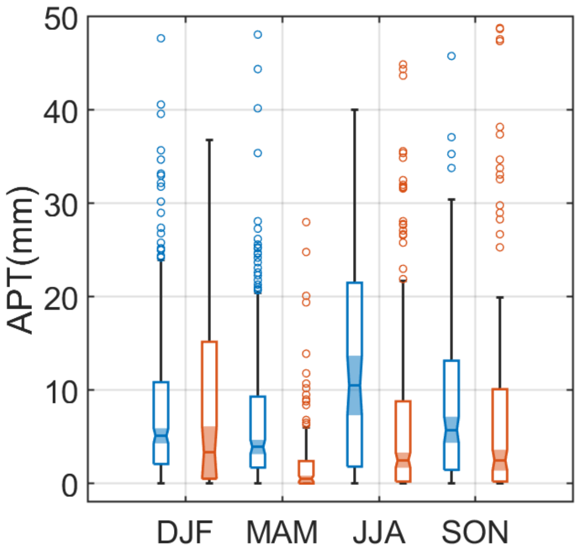

Figure S6. Box notch plot for each season of year 2017 comparing accumulated precipitation along trajectories (APT) for Clusters 1 (blue) and 2 (orange) from Fig. 4b. APT values were estimated from four-day HYSPLIT back trajectories reaching Bermuda (32.30° N, 64.77°W) at 100 m AGL. The middle, bottom, and top lines in each box represent the median, 25th percentile, and 75th percentile, respectively. Markers show extreme values identified based on 1.5×IQR (interquartile range) distance from the top of each box. Whiskers represent maximum and minimum values excluding extreme points. Boxes with notches and shaded regions that do not overlap have different medians at the 95% confidence level.

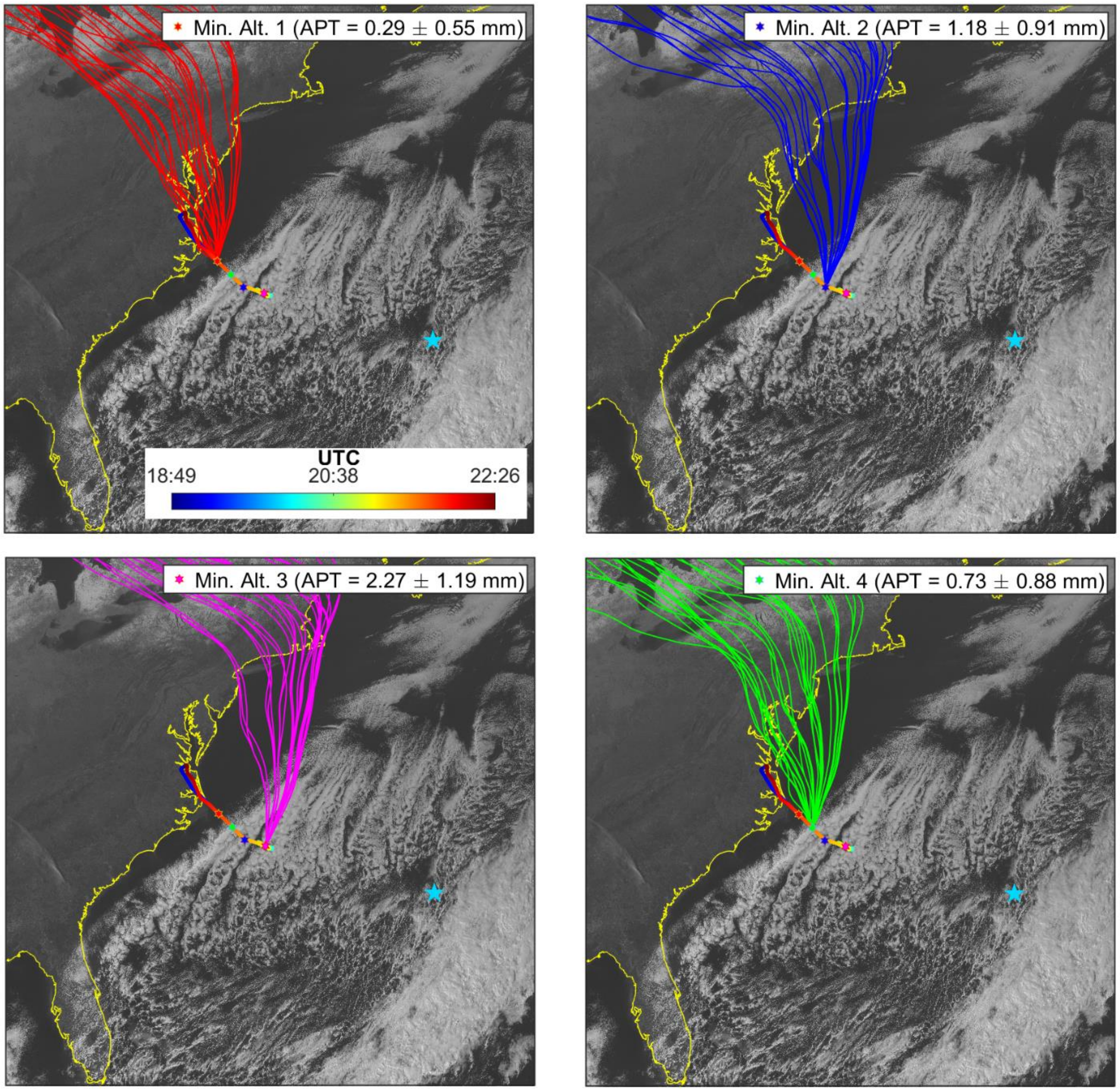

Figure S7. Trajectory ensembles for (a) Min. Alt. 1, (b) Min. Alt. 2, (c), Min. Alt. 3, and (d) Min. Alt. 4 legs, conducted by the HU-25 Falcon on 22 February 2020, overlaid on GOES 16 imagery obtained at 19:35:04 (UTC). The trajectory ensembles consisted of 27 individual trajectories obtained by offsetting the meteorological data by a fixed grid factor. Trajectory ensembles were calculated for the midpoints of four Min. Alt. legs, which are marked in each plot. The average (± standard deviation) of accumulated precipitation along the trajectory (APT) is also shown calculated for the recent history of the sampled air masses when they were over the ocean (time over land excluded from APT calculations).
